# ATR inhibition reverses the resistance of homologous recombination deficient MGMT^low^/MMR^proficient^ cancer cells to temozolomide

**DOI:** 10.18632/oncotarget.28090

**Published:** 2021-10-12

**Authors:** Lara H. El Touny, Curtis Hose, John Connelly, Erik Harris, Anne Monks, Angie B. Dull, Deborah F. Wilsker, Melinda G. Hollingshead, Michelle Gottholm-Ahalt, Sergio Y. Alcoser, Michael E. Mullendore, Ralph E. Parchment, James H. Doroshow, Beverly A. Teicher, Annamaria Rapisarda

**Affiliations:** ^1^Molecular Pharmacology Laboratory, Leidos Biomedical Research Inc., FNLCR, Frederick, MD, USA; ^2^Clinical Pharmacodynamic Biomarkers Program, Applied/Developmental Research Directorate, Leidos Biomedical Research Inc., FNLCR, Frederick, MD, USA; ^3^Biological Testing Branch, NCI, Frederick, MD, USA; ^4^In Vivo Evaluation Program, Leidos Biomedical Research Inc., FNLCR, Frederick, MD, USA; ^5^Division of Cancer Treatment and Diagnosis, NCI, Bethesda, MD, USA; ^6^Developmental Therapeutics Branch, Center for Cancer Research, NCI, National Institutes of Health, Bethesda, MD, USA; ^7^Molecular Pharmacology Branch, Developmental Therapeutics Program, NCI, Rockville, MD, USA; ^8^Current address: Division of Preclinical Innovation, National Center for Advancing Translational Sciences, National Institutes of Health, NIH, Bethesda, MD, USA

**Keywords:** TMZ, MMR, ATR, HR, REV3L

## Abstract

The therapeutic efficacy of temozolomide (TMZ) is hindered by inherent and acquired resistance. Biomarkers such as MGMT expression and MMR proficiency are used as predictors of response. However, not all MGMT^low/−ve^/MMR^proficient^ patients benefit from TMZ treatment, indicating a need for additional patient selection criteria. We explored the role of ATR in mediating TMZ resistance and whether ATR inhibitors (ATRi) could reverse this resistance in multiple cancer lines. We observed that only 31% of MGMT^low/−ve^/MMR^proficient^ patient-derived and established cancer lines are sensitive to TMZ at clinically relevant concentrations. TMZ treatment resulted in DNA damage signaling in both sensitive and resistant lines, but prolonged G_2_/M arrest and cell death were exclusive to sensitive models. Inhibition of ATR but not ATM, sensitized the majority of resistant models to TMZ and resulted in measurable DNA damage and persistent growth inhibition. Also, compromised homologous recombination (HR) via RAD51 or BRCA1 loss only conferred sensitivity to TMZ when combined with an ATRi. Furthermore, low REV3L mRNA expression correlated with sensitivity to the TMZ and ATRi combination *in vitro* and *in vivo*. This suggests that HR defects and low REV3L levels could be useful selection criteria for enhanced clinical efficacy of an ATRi plus TMZ combination.

## INTRODUCTION

Temozolomide (TMZ) is a DNA alkylating agent that is approved for standard-of-care of glioblastoma and is a clinical standard-of-care for advanced melanoma, respectively [1, 2]. TMZ is currently being evaluated in combination with other agents in >300 clinical trials targeting solid tumors (clinicaltrials.gov). Twenty five percent of these trials involve cancers unrelated to melanomas and gliomas specifically and brain cancers in general and include breast, pancreatic, colorectal as well as lung cancers, suggesting an increased interest in the evaluation of TMZ efficacy across a multitude of cancers. TMZ-induced DNA damage elicits many responses including apoptotic cell death, autophagy and senescence in cancer cells [3]. The TMZ-derived active species form methyl adducts on DNA bases, of which O^6^-methylguanine (O^6^-MeG) is the most deleterious [4]. Cells have the capacity to repair O^6^-MeG adducts through the activity of the suicide enzyme methylguanine-DNA methyltransferase (MGMT), thus mediating resistance to TMZ and related alkylating agents. In the absence of MGMT, O^6^-MeG acts as a miscoding template during replication and activates the DNA mismatch repair (MMR) pathway, which then induces replication fork arrest during DNA synthesis [5]. Stalling of the DNA replication fork progression, or replicative stress (RS), activates both DNA damage response (DDR) and DNA damage tolerance (DDT) pathways. Together DDR and DDT are pivotal for completing replication and preventing fork breakage, which averts the formation of cytotoxic double-strand breaks (DSBs) [6] and genomic instability [7].

DDR can promote the recovery of stalled replication forks through a regulated process primarily controlled by the replication checkpoint kinase ATR (ATM and rad3-related) [8]. Two critical outcomes of ATR activation are the inhibition of cell-cycle progression and suppression of late origin firing, events mediated by CHK1 (Checkpoint kinase 1) [8, 9]. Replication forks that are stabilized by ATR can be restarted after the removal of the stress, making ATR an attractive target for cancer therapy. Indeed, it has been shown that ATR inhibition is effective in killing cells with oncogene-induced replication stress [10]. Exposure to ATR inhibitors (ATRi) increased the toxicity of radiation [11] and chemotherapeutic agents such as platinums, gemcitabine [12, 13], and TMZ [14]. The prevention of prolonged fork stalling and completion of replication is also achieved through DDT, an error-prone system that allows cells to bypass DNA lesions either by using the sister chromatid as a template [8] or by incorporating nucleotides across from a lesion through a process known as translesion synthesis (TLS). Low-fidelity polymerases, such as Pol ζ, Pol κ, Pol ι, Pol η, and REV1, are key players in TLS and several reports indicate that their overexpression enhances DDR, resulting in resistance to DNA damaging therapy [15].

Two biomarkers, MGMT epigenetic silencing, which is correlated with TMZ responsiveness [16] and MMR deficiency, which is correlated with TMZ resistance in the absence of MGMT [5], have been evaluated clinically with limited success [6, 17]. This prompted us to investigate the role of the DDR pathway, and ATR specifically, in mediating TMZ resistance in MGMT^low/inhibited^/MMR^proficient^ models from various cancer types. We aimed to identify the molecular determinants that make TMZ-treated tumors more reliant on ATR and therefore more responsive to a combination of TMZ + ATRi.

By evaluating a panel of newly-established and well-characterized cell lines of various tissues of origin, we confirmed a poor association between lack of MGMT activity (either via absence of expression or chemical inhibition) in MMR-proficient models and sensitivity to TMZ at clinically-relevant concentrations. In the majority of the models tested from different cancer types, ATR-mediated responses were demonstrated to be pivotal for TMZ resistance. Finally, we identified HR deficiency (mediated by BRCA1 defects or RAD51 inhibition/knockdown) and alterations in DDT components (reduced REV3L mRNA expression) as determinants of sensitivity to the TMZ + ATRi combination *in vitro* and *in vivo*.

## RESULTS

### Persistent ATR activation and DSB signaling correlate with TMZ sensitivity in MGMT^low/−ve^/MMR^proficient^ models

**Figure 1:**
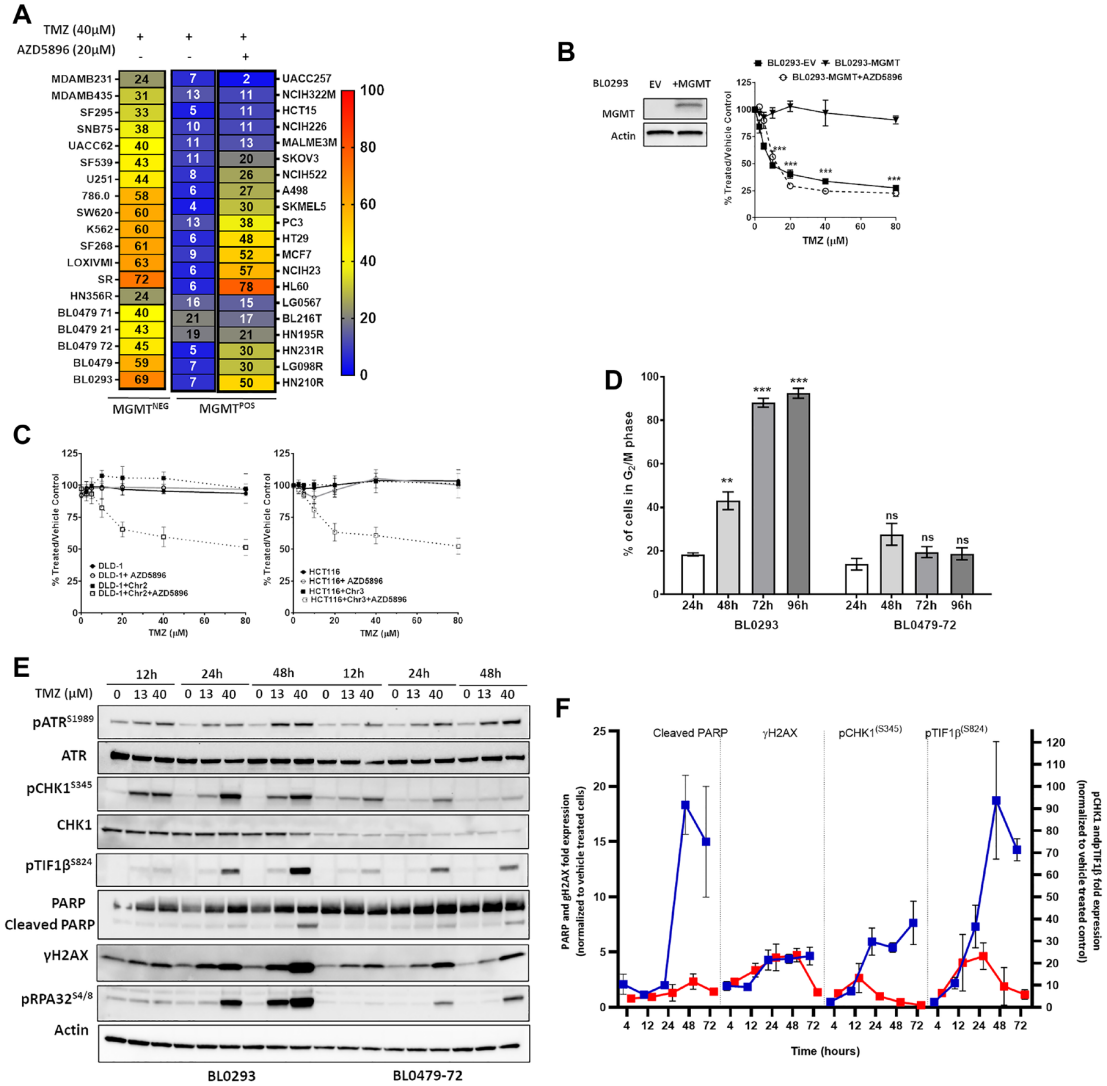
Response of cancer lines to temozolomide in the context of MGMT and MMR. (**A**) Thirty-nine cell lines were treated with TMZ (40 μM) for 96 h. MGMT^+ve^ cells were exposed to TMZ with or without AZD5896 (20 μM). Heat maps represent the mean percent growth inhibition of at least 3 independent experiments. (**B**) MGMT expression was verified by Western blotting (*left*) from lentivirally transduced BL0293-EV (empty vector control) and BL0293-MGMT. The cells were treated with increasing concentrations of TMZ for 96 h. BL0293-MGMT were exposed to TMZ with or without AZD5896 (20 μM). Percent proliferation relative to the vehicle control is plotted as the mean ± SEM of 2 independent experiments (^***^ *p* < 0.001 relative to BL0293 + MGMT). (**C**) DLD-1 and DLD-1 + Chr2 (*left*), HCT116 and HCT116 + Chr3 (*right*) were treated with increasing concentrations of TMZ for 96 h ± AZD5896 (10 μM). Percent proliferation relative to the vehicle control is plotted as the mean ± SEM of 3 independent experiments. (**D**) BL0293 and BL0479-72 were treated with TMZ (40 μM) and the cell cycle was analyzed at indicated time points. The percentage of cells in the G_2_/M phase are plotted as mean ± SEM from 2 independent experiments (compared to 24 h, ns (non-significant), ^***^ *p* < 0.001, ^****^ *p* < 0.0001). (**E**) BL0293 and BL0479-72 were treated with TMZ for the indicated time points. The activation and expression of DDR pathway components were assessed by Western blot and the average fold expression ± SEM of pCHK1, pTIF1β, Cleaved (Cl.) PARP and γH2AX in lysates of BL0293 (blue lines) and BL0479-72 (red lines) treated with TMZ (40 μM) over vehicle treated cells at each time point has been quantified from at least 3 experiments repeats and shown in (**F**).

Low MGMT expression is a recognized determinant of TMZ sensitivity, although resistance among MGMT^−ve^ GBM patients is observed [17]. A panel of 12 PDCs and 27 NCI60 lines with varied TP53/MGMT status (Supplementary Tables 1 and 2, Supplementary Figure 1A) [14] was treated with TMZ (in the presence or absence of the pseudo-substrate MGMT inhibitor AZD5896). We observed that 12 of 39 MGMT^−ve/inhibited^ lines exhibited a substantial reduction in cell growth (≥ 50%) calculated as 100-percent [luminescence of TMZ-treated cells/vehicle treated cells] or (100- %T/V) upon treatment with a clinically-relevant TMZ concentration (40 μM; C_max_ 37.6 μM [18]), whereas all MGMT^+ve^ lines were resistant to TMZ in the absence of AZD5896, confirming that MGMT expression confers resistance to TMZ (Figure 1A). Indeed, lentiviral-mediated MGMT expression in TMZ-sensitive BL0293 rendered the model resistant to TMZ (up to 80 μM) and sensitivity was restored by pre-treatment with AZD5896 (Figure 1B); however, low/inhibited MGMT status was not sufficient to account for the TMZ response by all cell lines.

Deficiency in MMR, a regulator of replication fidelity, has been associated with TMZ resistance [6]. Indeed, the MMR-deficient cell lines SKOV3 and HCT15 (Figure 1A) and DLD-1 and HCT116 (Figure 1C) were resistant to TMZ ± AZD5896. Restoration of MMR in DLD-1 via supplementation of Chr2 (for MSH2/6 expression) and HCT116 via supplementation of Chr3 (for MLH1 expression) (Supplementary Figure 1B) sensitized both cell lines to TMZ + AZD5896, but did not reduce the cell number ≥ 50% (Figure 1C). Thus, low/inhibited MGMT and functional MMR are necessary, but not sufficient, to confer TMZ sensitivity.

To elucidate additional factors involved in the TMZ sensitivity of MGMT^−ve^/MMR^proficient^ models, cell cycle profiles of MGMT^−ve^ BL0293 (sensitive) and BL0479-72 (insensitive) bladder cancer lines were compared after exposure to TMZ (40 μM). BL0293 arrested in the G_2_/M phase, reaching 88.1% by 72 h posttreatment. In contrast, BL0479-72 exhibited a transient accumulation in the G_2_/M phase with a peak of 27.6% at 48 h before decreasing to 19.5% at 72 h and 96 h post-TMZ exposure (Figure 1D). Thus, TMZ-induced DNA damage to BL0479-72 was either insufficient to cause sustained cell cycle arrest or it was rapidly resolved. Western analysis revealed a robust and more pronounced TMZ-induced DNA damage signature in sensitive BL0293 cells compared to resistant BL0479-72 cells. DSB signaling occurred early in BL0293 cells with γH2AX, an early cellular marker of DSB, pATM^S1981^ and pTIF1β^S824^ appearing at 4 h and maintained thereafter, while PARP cleavage was observed 48 h posttreatment (Supplementary Figure 1C and 1D). ATR activation (pATR^S1989^) was significantly induced at 12 h and sustained up to 72 h, whereas pCHK1^S345^ peaked at 24 h and remained elevated thereafter (Figure 1E–1F and Supplementary Figure 1C and 1D). In contrast, γH2AX peaked in BL0479-72 cells 24 h after TMZ treatment and decreased by 72 h. ATR-dependent pCHK1^S345^ induction was also transient, peaking at 12 h and subsequently decreasing. Importantly, no significant PARP cleavage was observed through 72 h (Figure 1E–1F and Supplementary Figure 1E–1F) or 96 h (data not shown). Similarly, the TMZ-resistant LG0567 non-small cell lung carcinoma cell line exhibited a transient G_2_/M accumulation and activation of DDR upon exposure to TMZ + AZD5896 with no evidence of PARP cleavage (Supplementary Figure 2A–2B). Transient activation of DDR signaling in TMZ-resistant cell lines suggested a faster ATR-driven resolution of damage compared with TMZ-responsive lines and further suggested that ATR inhibition might increase TMZ sensitivity in MGMT^−ve^/MMR^proficient^ models.

### Inhibiting ATR in combination with TMZ promotes sustained DNA damage, growth inhibition and cell death in a subset of MGMT^−ve^/MMR^proficient^ models

**Figure 2:**
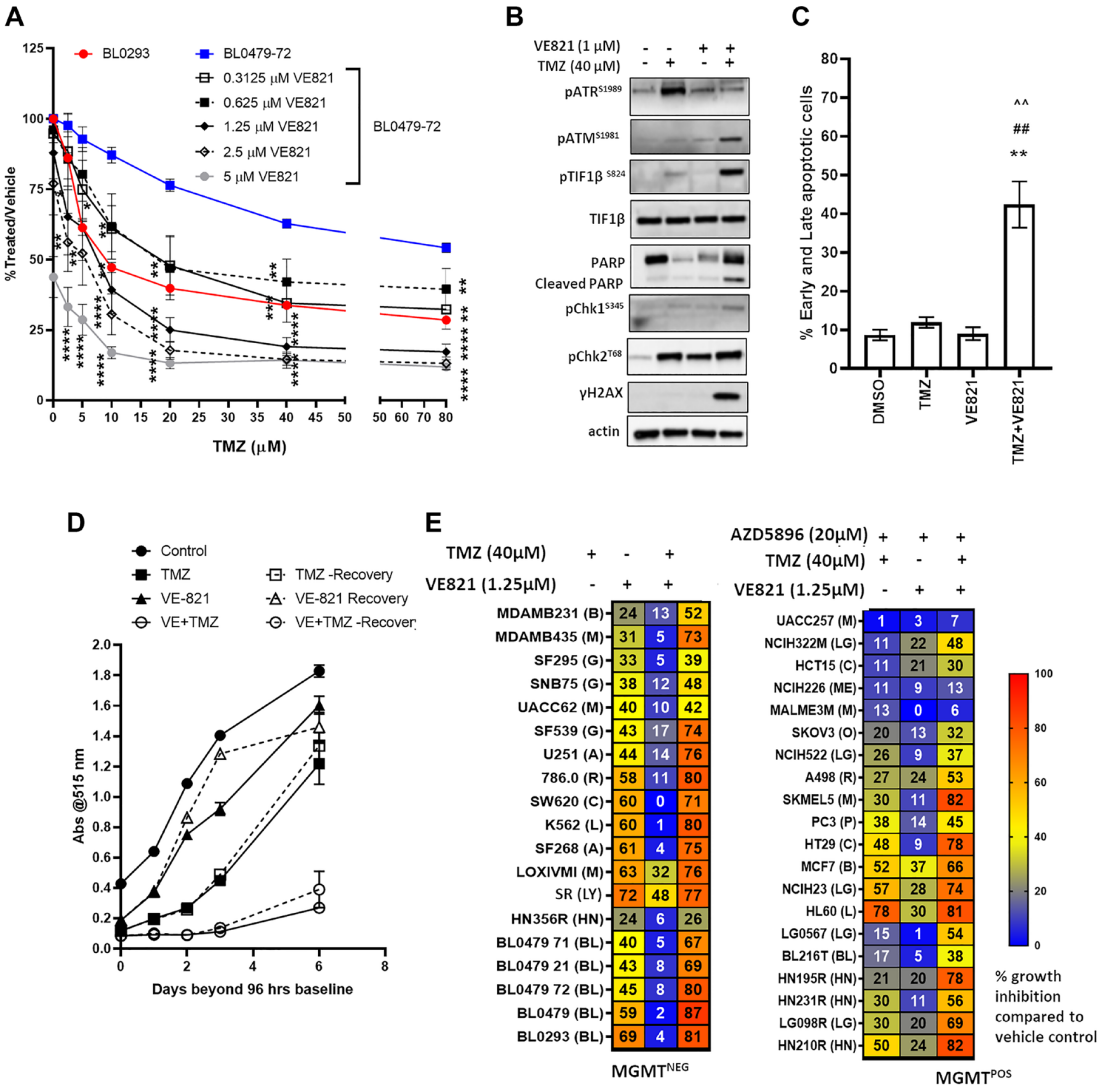
ATR inhibition, in combination with TMZ, induces persistent DNA damage, growth inhibition and cell death in the majority of MGMT^−ve^/MMR^proficient^ models. (**A**) BL0479-72 cells were exposed to 6 concentrations of TMZ in combination with 5 concentrations of VE821 for 96 h (*left*), while BL0293 cells were treated with the same 6 concentrations of TMZ only. Results were plotted as line graphs with average % T/V calculated as 100× (blanked luminescence of wells at each dose/blanked luminescence of vehicle control wells), ^*^ *p* < 0.05, ^**^ *p* < 0.01, ^***^ *p* < 0.001, ^****^ *p* < 0.0001 compared to same dose of TMZ as a single agent (**B**) BL0479-72 cells were treated with vehicle or TMZ (40 μM) ± VE821 (1 μM) for 48 h and DDR pathway components were assessed by Western blot (**C**) Early and late apoptosis (Annexin V + PI positive cells) was measured via Annexin V^+ve^/PI^+ve^ staining after BL0479-72 cells were treated with vehicle or TMZ (40 μM) ± VE821 (1 μM) for 72 h. The percentage of early and late apoptotic cells are plotted as mean ± SEM of 3 independent experiments (*p* < 0.01 compared to ^**^DMSO, ^##^TMZ, or ^^^^VE821). (**D**) BL0479-72 were treated with vehicle or TMZ (40 μM) ± VE821 (0.5 μM) for 96 h. The treatments were either removed after 96 h and replaced with inhibitor-free medium (Recovery) or retained for an additional 6 days. Proliferation was assessed by SRB at the indicated days beyond 96 h and plotted as mean ± SEM for 3 independent experiments. (**E**) Thirty-nine cell lines were treated with TMZ (40 μM) ± VE821 (1.25 μM) for 96 h. The 20 MGMT^+ve^ cells were also treated with AZD5896 (20 μM). Heat maps indicate the average percent growth inhibition from at least 3 independent experiments. Abbreviations B: breast cancer; M: melanoma; G: glioblastoma; A: astrocytoma; R: renal cancer; L: leukemia; LY: lymphoma; LG: lung cancer; HN: head and neck cancer; BL: bladder cancer; ME: mesothelioma; O: ovarian cancer; P: prostate cancer.

ATR, but not ATM, is necessary for the G_2_/M arrest induced by clinically-relevant TMZ concentrations [19]. To determine whether ATR inhibition plays a role in the TMZ resistance of MGMT^−ve^/MMR^proficient^ models, BL0479-72 was treated with TMZ in combination with the ATRi VE821. VE821 sensitized BL0479-72 cells to TMZ, resulting in a TMZ IC_50_ < 20 and IC_50_ < 10 μM with 0.625 and 1.25 μM VE821, respectively, with significant differences seen as early as the 2.5 μM TMZ dose + 1.25 μM VE821 compared to 2.5 μM TMZ alone (*p* < 0.05) and mimicking the growth inhibition achieved by TMZ alone in BL0293 cells (Figure 2A). The single agent VE821, while not severely affecting BL0479-72 proliferation at 1 μM, reduced ATR activation (pATR^S1981^) in the presence of TMZ (Figure 2B). Combination-treated BL0479-72 exhibited induction of γH2AX, pTIF1β^S824^ and PARP cleavage at 48 h (Figure 2B). At 72 h, apoptosis, measured by AnnexinV/PI staining, was detected in 42.4 ± 5.9% combination-treated BL0479-72 cells compared to cells treated with vehicle (8.7 ± 1.4%), TMZ (11.9 ± 1.4%) and VE821 (9.0 ± 1.6%) (Figure 2C). A similar sensitization was observed by combining TMZ with other ATRi, AZD6738 and VE822 (also known as VX970 or M6620) (Supplementary Figure 3A–3C), but not the ATM inhibitor KU55933 (Supplementary Figure 3D). The inhibition of ATR and induction of apoptosis by TMZ with other ATRi is shown in Supplementary Figure 3E–3F. Interestingly, as evidenced by the densitometry of ATR activation in Supplementary Figure 3D, complete inhibition of ATR is not necessary to sensitize TMZ-resistant cells to physiologically achievable levels of TMZ, which was achieved with ~50% inhibition of ATR.

To evaluate whether combining TMZ with an ATRi sustains growth inhibition, BL0479-72 were treated with TMZ for 96 h and followed for an additional 6 days. Exposure of BL0479-72 cells to TMZ (40 μM) or VE821 (0.5 μM) for 96 h resulted in a 34% and 13% reduction in cell numbers, respectively, but growth was observed in the viable fractions for the duration of the experiment, confirming resistance to TMZ as a single agent. At 96 h the combination of TMZ + VE821 decreased BL0479-72 cell numbers by 85% and sustained growth inhibition of the remaining viable fraction for 6 days following drug removal (Figure 2D). Similar results were obtained with VE822 and AZD6738 (Supplementary Figure 3G), suggesting that TMZ in combination with ATRi causes a prolonged inhibition of proliferation that is not observed with either drug/inhibitor alone.

Several PDC and NCI60 lines (17 MGMT^−ve^ and 20 MGMT^+ve^) were evaluated with TMZ + VE821, (AZD5896 (20 μM) was included for all 20 MGMT^+ve^ lines). Twenty-seven of these models were classified as TMZ-resistant, defined as < 50% inhibition with 40 μM TMZ at 96 h, while 12 models were classified as TMZ-sensitive, defined as > 50% inhibition with 40 μM TMZ at 96 h. ATR inhibition sensitized 14 resistant models (52%) and increased the sensitivity of 9 moderately sensitive models (Figure 2E). However, ATR inhibition did not overcome the TMZ resistance mediated by MMR deficiency in SKOV3 and HCT15. The requirement of MMR proficiency for TMZ sensitization by ATRi was confirmed in DLD-1 and HCT116 cells. MMR restoration sensitized these cell lines to TMZ + ATRi, with TMZ IC_50_ values of 10 μM and < 2.5 μM, respectively (Supplementary Figure 4). Furthermore, no sensitization to TMZ + ATRi was observed in MGMT^+ve^ lines in the absence of AZD5896 (data not shown), demonstrating that ATR protects against O^6^-MeG cytotoxicity as reported [20] and confirming the recent report that MGMT^+ve^ cells glioblastoma cells are not sensitized to TMZ by ATR inhibition [21].

### Inhibition of several ATR functions is necessary to induce cytotoxicity in TMZ-treated BL0479-72 cells

ATR stabilizes stalled replication forks to avoid their collapse into DSBs and prevents mitotic entry via CHK1 and the G_2_/M checkpoint to avoid genomic instability [14]. Forty-eight hours of TMZ exposure resulted in a 9-fold increase in cells positive for γH2AX in TMZ-sensitive BL0293 versus a 5-fold increase in TMZ-resistant BL0479-72 (Supplementary Figure 5). The combination of VE821 (1 μM) with TMZ (40 μM) did not alter γH2AX accumulation in BL0293 cells but induced a 12-fold increase in BL0479-72 cells, compared to the vehicle-treated control. This suggests that the TMZ + ATRi combination causes replication fork collapse in the TMZ-resistant BL0479-72 cells.

**Figure 3:**
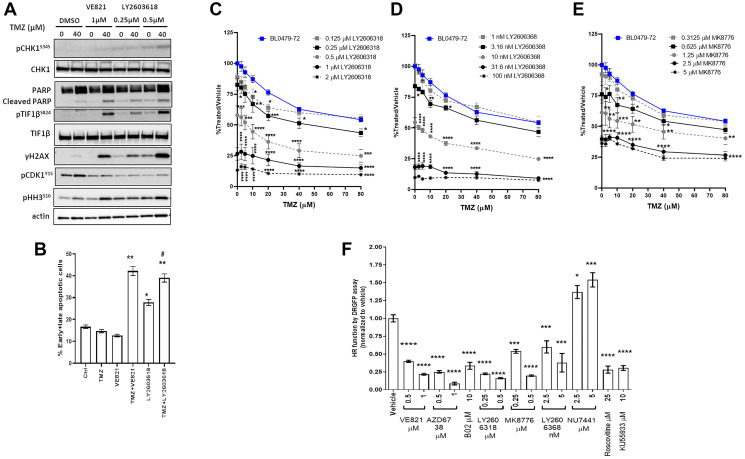
Similar to ATR inhibition, CHK1 inhibition induces DSB signaling, decreases HR-dependent DNA repair and sensitizes cells to TMZ. (**A**–**B**) BL0479-72 cells were treated with vehicle or TMZ ± VE821 or ± LY2606318 for 72 h and assessed for (A) induction of DNA damage, PARP cleavage and mitotic entry by Western blot and (B) early and late apoptosis by Annexin V^+ve^ and PI^+ve^ staining, respectively. The results are plotted as mean ± SEM of two independent experiments (^*^ *p* < 0.05, ^**^ *p* < 0.01 compared to DMSO, TMZ or VE821; ^#^ *p* < 0.05 compared to LY2606318). (**C**–**E**) Growth inhibition of BL0479-72 after combination treatment with TMZ and (C) LY2606318 (D) LY2606368 and (E) MK8776. Results were plotted as line graphs with average % T/V calculated as 100× (blanked luminescence of wells at each dose/blanked luminescence of vehicle control wells), ^*^ *p* < 0.05, ^**^ *p* < 0.01, ^***^ *p* < 0.001, ^****^ *p* < 0.0001 compared to same dose of TMZ as a single agent. (**F**) The HR function of BL0479-72 cells was measured by the DRGFP assay following a 48 h treatment with inhibitors of ATR (VE821 and AZD6738), CHK1 (LY2606318, MK8776, LY2606368), RAD51 (B02), DNAPK (NU7441), ATM (KU55933) and pan-CDK (roscovitine). Results are plotted as the mean ± SEM of at least 2 independent experiments (^*^ *p* < 0.05, ^***^ *p* < 0.001, ^****^ *p* < 0.0001).

CHK1 is an important mediator of ATR functions; therefore, we assessed whether CHK1 inhibitors (CHKi) recapitulate the effects of ATRi in combination with TMZ. The combination of TMZ and CHK1i (LY2603618, LY2606368, or M8776) at doses that induced pCHK1^S345^, a proposed biomarker of CHK1 inhibitor activity [22], induced pTIF1b^S824^, γH2AX and PARP cleavage in BL0479-72 cells, similar to TMZ + VE821 (Figure 3A and Supplementary Figure 6A). Also, the induction of apoptosis in BL0479-72 cells was similar for combinations of TMZ with VE821 or LY2603618 at 72 h (Figure 3B). The pCDK1^Y15^ reduction (indicating CDK1 activation and G_2_/M transition) and pH3^S10^ induction (a marker of chromosomal condensation during mitosis) concomitant with apoptosis suggests that ATR/CHK1 inhibition results in a bypass of the transient arrest seen in TMZ-treated BL0479-72 cells and the induction of mitotic catastrophe. Interestingly the combinations of TMZ + CHK1i did not exhibit the strong synergy observed with TMZ + VE821 (Figure 3C–3E and Supplementary Figure 6B for 3D response plots). This could be due to the concentration-dependent DNA damage and apoptosis observed with CHK1i alone, but not with ATRi (Figure 3A–3B). Alternatively, CHK1 might not be mediating all the functions of ATR upon TMZ treatment.

The role of the ATR-mediated G_2_/M checkpoint in TMZ resistance was further assessed in BL0479-72 cells by combining TMZ with the Wee-1 inhibitor MK-1775, since Wee-1 acts as a negative regulator of the G_2_/M transition by phosphorylating and inhibiting CDK1 [23]. Concentrations of MK-1775 that decrease pCDK1^Y15^ (0.25 and 0.5 μM, Supplementary Figure 6C) didn’t alter the response of BL0479-72 cells to TMZ (40 μM) beyond single agent activity (Supplementary Figure 6D), demonstrating that G_2_/M checkpoint abrogation alone cannot account for the response to TMZ + ATRi.

The genotoxicity of TMZ-induced lesions is mainly mediated by DSBs that are repaired by non-homologous end joining (NHEJ) and homologous recombination (HR). Repair of TMZ-mediated DSBs in late S/G_2_ phase is attributed to HR [24]. NHEJ inhibition by DNAPK inhibitors (NU7441 and NU7026) did not sensitize BL0479-72 to TMZ (Supplementary Figure 6E–6F) beyond the effect of the DNAPKi alone; therefore, the HR capacity of TMZ-sensitive and resistant bladder cancer lines was assessed using the DRGFP reporter [25]. The HR capacity of BL0293 and BL0479-72 was 9- and 4-fold lower, respectively, than HR-proficient HeLa cells (*p* < 0.0001, Supplementary Figure 6G). Inhibition of ATR or CHK1 reduced the BL0479-72 HR capacity dose-dependently (Figure 3F), similar to the pan-CDK inhibitor roscovitine and the ATM inhibitor KU55933. This suggests that sensitization to TMZ by ATR/CHK1 inhibition could be partly due to reduced HR. Inhibiting ATR had a greater impact on HR capacity than inhibiting Rad51, a major player in homologous strand exchange, a key step in the HR-mediated DNA repair, by a small molecule inhibitor B02 [26] (Figure 3F), supporting the notion that ATR controls several components of HR [8, 27].

### HR and TLS deficiencies sensitize cancer cells to the TMZ + ATRi combination

**Figure 4:**
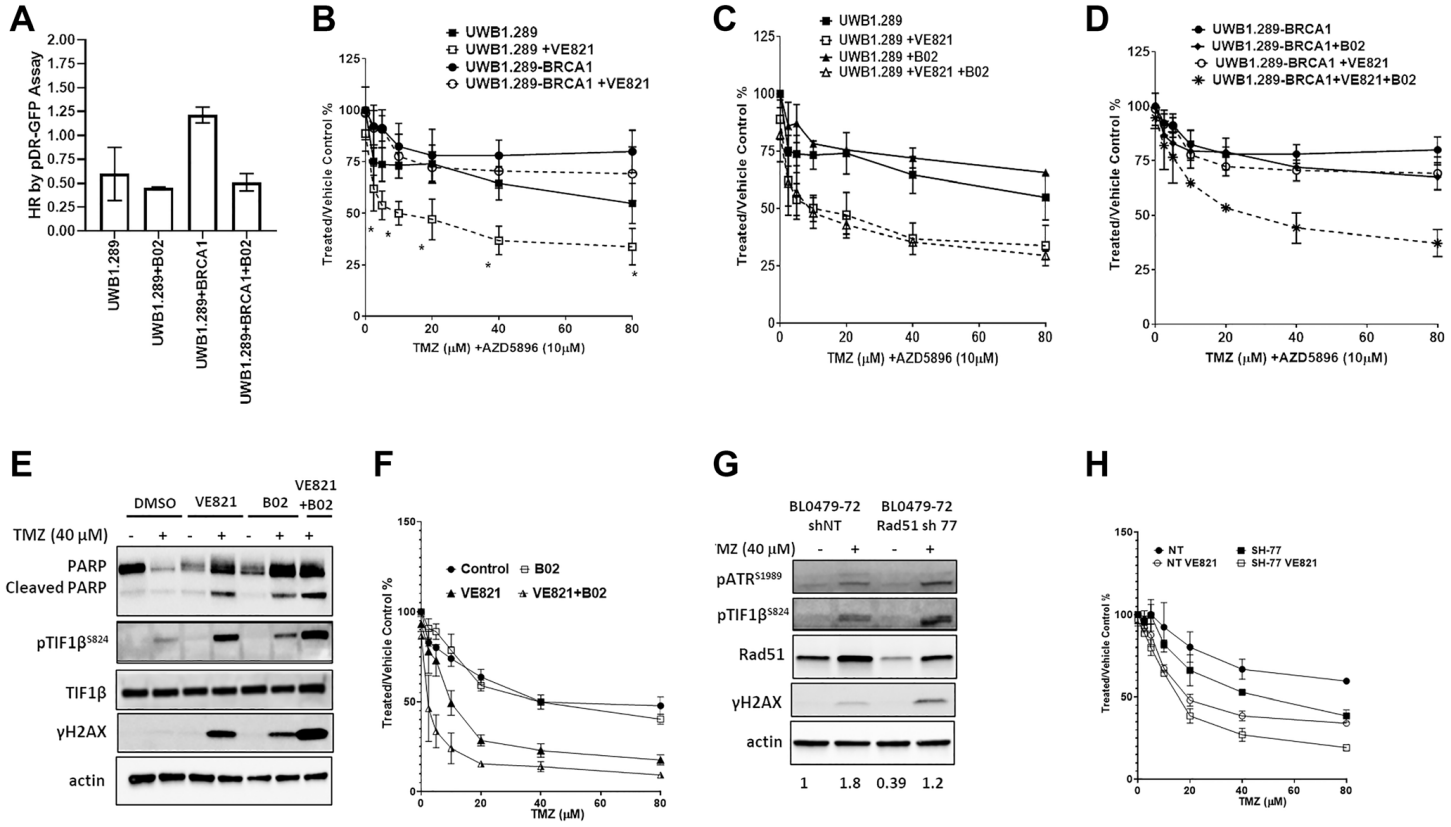
HR deficiencies sensitize cancer cell lines to the combination of TMZ and ATRi. (**A**) Homologous recombination capacity of UWB1.289 ± BRCA1 cells by the DRGFP assay. B02 was used at 2.5 μM. The results are shown as mean ± SEM of two independent experiments. (**B**) UWB1.289 and UWB1.289-BRCA1 cells were exposed to increasing concentrations of TMZ (in the presence of 10 μM AZD5896) ± VE821 (0.5 μM) for 96 h. Cell proliferation relative to the vehicle control is plotted as the mean ± SEM of two independent experiments (^*^ *p* < 0.05 compared to UWB1.289-BRCA1 + VE821). (**C**) UWB1.289-BRCA1 cells and (**D**) UWB1.289 cells were exposed to increasing concentrations of TMZ (in the presence of AZD5896, 10 μM) ± VE821 (0.5 μM) and ± B02 (2.5 μM) for 96 h. Cell proliferation relative to the vehicle control is plotted as the mean ± SEM of two independent experiments (^*^ *p* < 0.05 compared to UWB1.289-BRCA1 + VE821). (**E**) BL0479-72 cells were treated with vehicle or TMZ (40 μM) ± VE821 (1 μM) ± B02 pre-treatment (1 h, 10 μM) and exposed for 72 h before assessing DDR signaling by Western blot. (**F**) BL0479-72 were treated with increasing concentrations of TMZ ± B02 pre-treatment (1 h, 10 μM) and VE821 (1.25 μM) and exposed for 96 h. Cell proliferation relative to the vehicle control is plotted as the mean ± SEM of two independent experiments. (^*^ *p* < 0.05 compared to VE821). (**G**) BL0479-72 cells stably expressing non-target (NT) shRNA or RAD51 shRNA 77 (SH-77) were treated with TMZ. After 72 h DDR signaling was assessed by Western blot. Densitometry was used to quantify RAD51 expression and the values from each condition were averaged from the two experiments. The ratio of RAD51 expression relative to the control (vehicle + shNT) is shown for each condition at the bottom of the figure. (**H**) BL0479-72 cells stably expressing NT shRNA or SH-77 were treated with increasing concentrations of TMZ ± VE821 (1.25 μM) for 96 h. Cell proliferation relative to the vehicle control is plotted as the mean ± SEM of two independent experiments.

To evaluate the role of HR in responses to TMZ + ATRi, the BRCA1-null human ovarian cancer line UWB1.289 and its BRCA1-supplemented sub-line (UWB1.289-BRCA1) were examined. By virtue of BRCA1’s role in HR and its association with Rad51, as expected, BRCA1 supplementation resulted in a 2-fold increase in HR capacity compared to the BRCA1-null UWB1.289 cells which was reduced after treatment with B02 (2.5 μM) (Figure 4A). The induction of γH2AX and pTIF1b^S824^ in both models treated with TMZ + AZD5896 (Supplementary Figure 7) indicates DSB signaling; however, cell growth was not significantly affected (IC_50_ > 80 μM) (Figure 4B). The addition of VE821 (0.3125 μM, a concentration that does not affect growth as a single agent) to TMZ + AZD5896 sensitized UWB1.289 cells (TMZ IC_50_ = 10 μM) and resulted in PARP cleavage, which was not observed in UWB1.289-BRCA1 cells (Figure 4B and Supplementary Figure 7). Thus, decreased HR capacity is a determinant of sensitivity to the TMZ + ATRi combination, but not TMZ alone. Supporting this, RAD51 inhibition sensitized UWB1.289-BRCA1, but not UWB1.289, to TMZ + VE821 treatment (Figure 4C–4D, TMZ IC_50_ = 25.8 μM), which coincided with PARP cleavage and evidence of mitotic entry (reduced pCDK1^Y15^ and increased pHH3^S10^) suggesting mitotic catastrophe (Supplementary Figure 7). To confirm that HR deficiencies mediate sensitivity to the TMZ + ATRi combination, HR was further compromised in BL0479-72 cells by either B02 pre-treatment or shRNA-mediated RAD51 knockdown. B02 treatment resulted in a 3-fold reduction in HR capacity (Figure 3F) and moderate activation of DSB signaling (pTIF1β^S824^ and γH2AX, Figure 4E); however, no significant change in sensitivity to TMZ was observed (Figure 4F). Likewise, RAD51 knockdown in BL0479-72 (resulting in a 61% and 33% reduction of endogenous and TMZ-induced RAD51, respectively) did not sensitize cells significantly to clinically-relevant concentrations of TMZ (IC_50_ > 40 μM), despite the induction of pTIF1β^S824^ and γH2AX (Figure 4G–4H). However, genetic and pharmacological inhibition of RAD51 further sensitized BL0479-72 cells to TMZ + VE821 compared to control cells (Figure 4H), in line with the notion that HR deficiencies increase reliance on ATR activity in cancer cells [28].

DNA damage tolerance mechanisms, specifically TLS DNA polymerases, play a significant role in bypassing O^6^-MeG-mediated replication blocks [29]. Moreover, TLS polymerases have been implicated in *de novo* or acquired resistance to alkylating and chloro-ethylating agents [30, 31]. Specifically REV3L, the catalytic subunit of pol ζ, is thought to be one of the major components of error-prone TLS and plays a significant role in the chemoresistance of many cancers [32, 33]. On the other hand, ribonucleotide reductase (RRM2) has been associated with TMZ resistance in melanoma cells *in vitro* [34].

**Figure 5:**
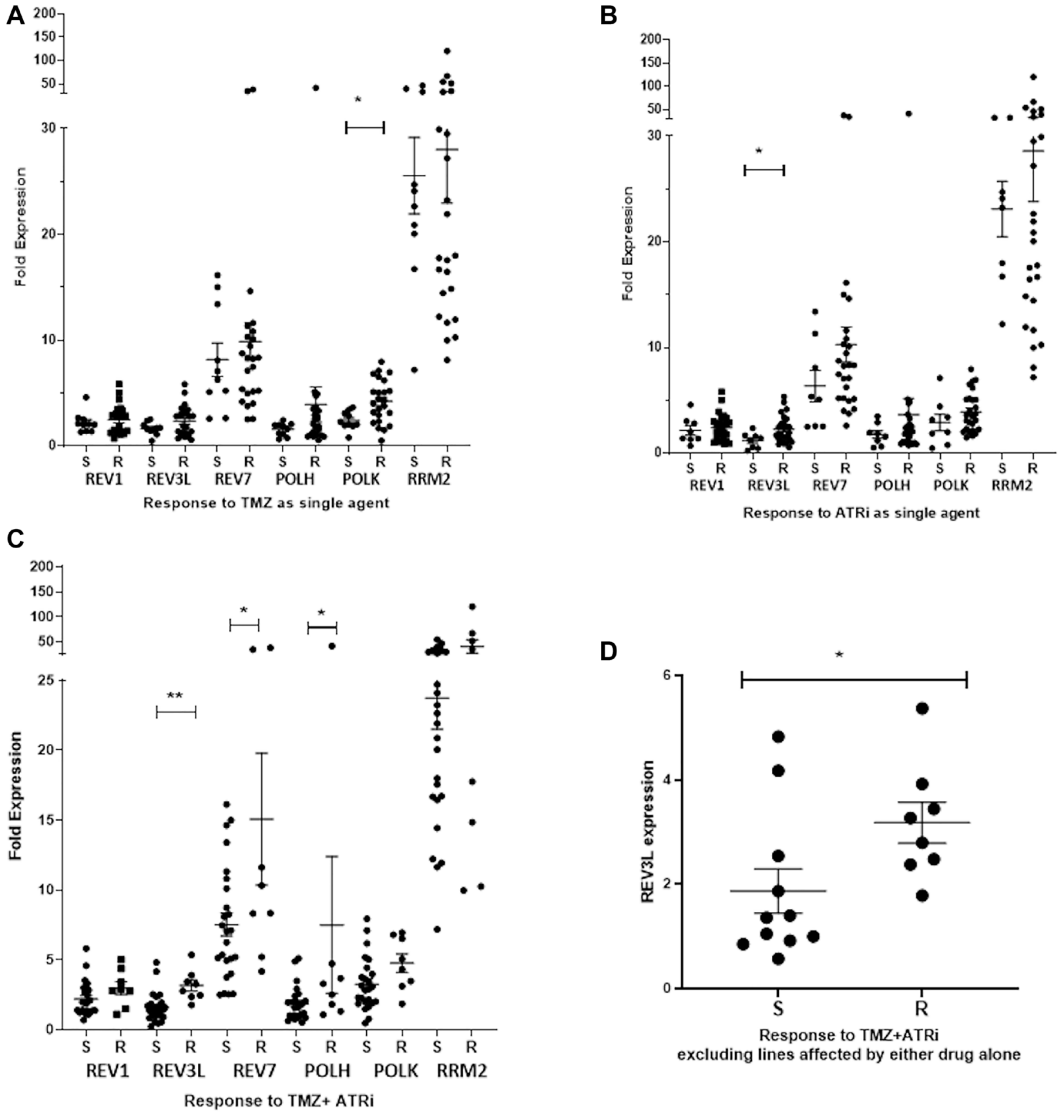
Low REV3L mRNA expression is associated with sensitivity to the combination of TMZ + ATRi. The mRNA expression of TLS polymerases and RRM2 was evaluated by qPCR. The values were calculated as 2^-[GOI Cts-GAPDH Cts]*10^3^) from qPCR data (GOI: gene of interest). (**A**) The expression of TLS polymerases and RRM2 in models sensitive (S, GI ≥ 50%) and resistant (R, GI < 50%) to TMZ. (**B**) The expression of TLS polymerases and RRM2 in models sensitive (S, GI ≥ 20%) and resistant (R, GI < 20%) to VE821. (**C**) The expression of TLS polymerases and RRM2 in models sensitive (S, GI ≥ 50%) and resistant (R, GI < 50%) to TMZ + VE821. (**D**) The expression of REV3L in models not sensitive to TMZ or ATRi as single agents but sensitized by TMZ + ATRi. Mean expression is reported ± SEM with ^*^ *p* < 0.05, ^**^ *p* < 0.01 by an unpaired *t*-test.

Next we investigated whether TLS polymerase expression affects responses to the combination of TMZ + ATRi. MGMT^−ve/inhibited^ cell lines were divided into groups based on the extent of growth inhibition after treatment with TMZ (40 μM) alone or in combination with VE821 (1.25 μM). Cell lines with a growth inhibition (GI) >50% for TMZ alone or in combination with VE821 were classified as sensitive, whereas those with a GI <50% were considered resistant. Cell lines with a GI >20% were classified as sensitive to VE821, whereas those with a GI <20% were considered resistant. POLK mRNA expression was significantly lower in TMZ-sensitive lines compared to TMZ-resistant cells (*p* < 0.05, Figure 5A), while REV3L was lower in VE821 sensitive lines (*p* < 0.05, Figure 5B). Sensitivity to TMZ + VE821 was associated with significantly lower REV3L, REV7 and POLH expression (*p* < 0.01, *p* < 0.05 and *p* < 0.05, respectively) (Figure 5C). Analysis of TLS polymerase expression in models that were not sensitive to TMZ and VE821 as single agents showed an association between low REV3L mRNA expression and sensitivity to TMZ + VE821 (*p* < 0.05, Figure 5D), suggesting that low REV3L mRNA expression can be a pre-determinant of improved response to TMZ + ATRi.

### MGMT^low^/MMR^proficient^/HR^deficient^ models are sensitive to TMZ + ATRi *in vivo*

**Figure 6:**
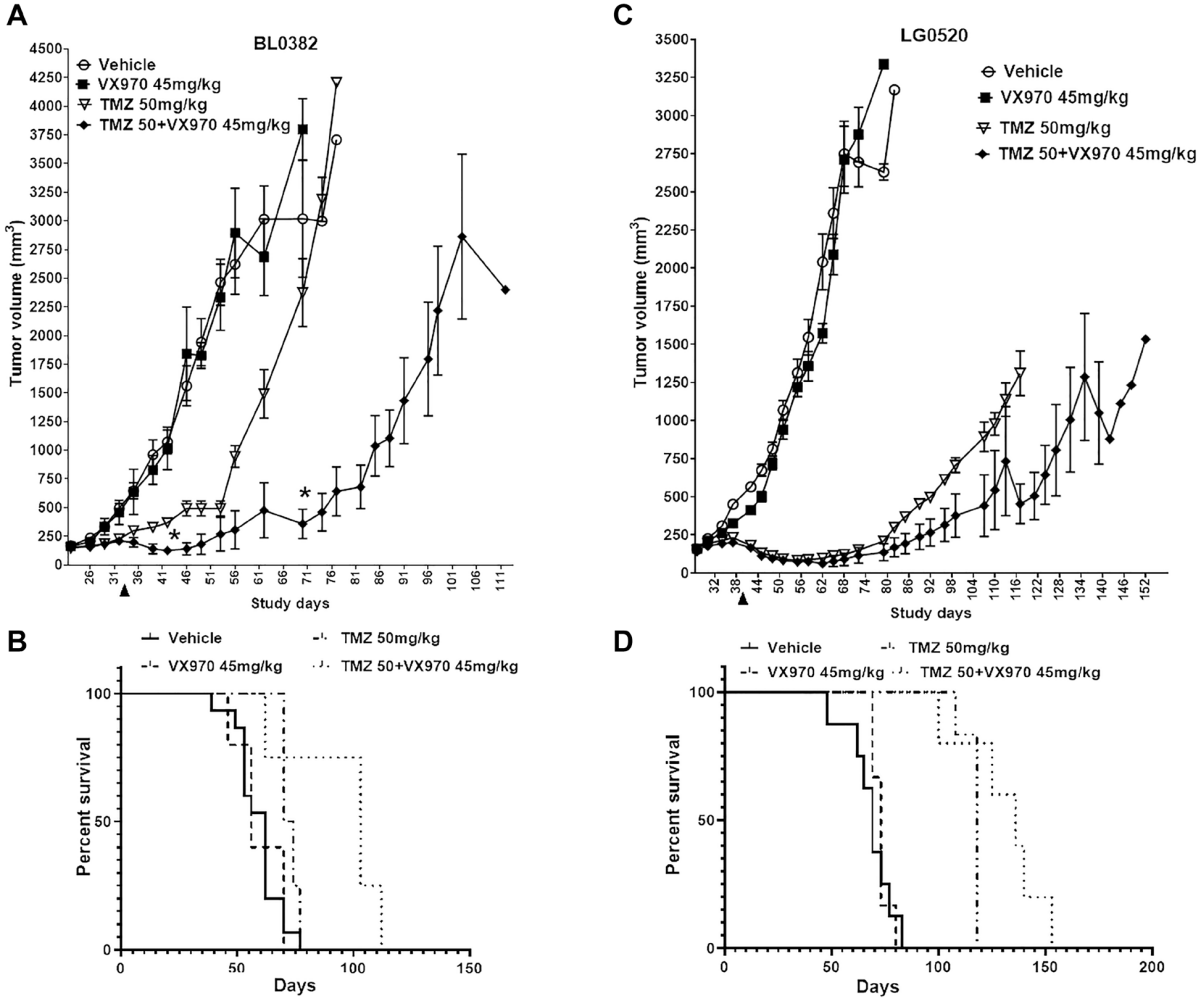
MGMT^low^/MMR^proficient^/LOH^high^ PDX models are responsive to the TMZ + ATRi combination. Mice harboring (**A**–**B**) BL0382 or (**C**–**D**) LG0520 tumor fragments were treated for 2 cycles with vehicle, TMZ (50 mg/kg; QDx5, 2 days rest), VE822 (45 mg/kg; QDx4, 3 days rest) or TMZ + VE822. Treatments were administered between days 22 and 33 (BL0382) or days 28 and 39 (LG0520). (A, C) Mean tumor volume ± SD is shown (^*^ *p* < 0.05 by the Holm-Sidak method). (B–D) Survival graphs for PDXs (B) BL0382 and (D) LG0520. Arrowheads depict the end of treatment.

Our *in vitro* results indicated that MGMT^low^ MMR^proficient^ HR^deficient^ cancer cells with low REV3L mRNA expression are sensitive to the TMZ + ATRi combination. To examine this further, we identified 2 models from the NCI Patient-Derived Models Repository (PDMR) with low MGMT, MMR proficiency, HR deficiency (defined as LOH>16%) [35]) and either low REV3L mRNA expression (BL0382) or high expression (LG0520, Supplementary Figure 8A). Mice implanted with BL0382 or LG0520 tumor fragments were treated orally with 2 cycles of vehicle, TMZ 50 mg/kg (QDx5, 2 days rest), VE822 45 mg/kg (QDx4, 3 days rest) or TMZ + VE822 and followed for 111 and 152 days, respectively. The BL0382 and LG0520 tumors were not sensitive to VE822 and both models showed some response to TMZ (a cytostatic response during treatment for BL0382 (days 22–33) and tumor regression posttreatment for LG0520 (after day 39, Figure 6A–6B). Mice bearing LG0520, but not BL0382, had increased survival in the TMZ-only group versus vehicle (Figure 6C–6D). The TMZ + VE822 combination resulted in LG0520 tumor growth reductions in 4 out of 6 mice (tumors < 200 mm^3^) for 53 days posttreatment-end, compared to 40 days in the TMZ group (Supplementary Figure 8C), and a survival advantage of 18 days compared to TMZ-only (Figure 6D). On the other hand, BL0382 tumors insensitive to TMZ as a single agent were significantly reduced in volume by 85% at days 70 and 74 after administration of TMZ + VE822 compared to TMZ alone, with 3 out of 4 mice exhibiting tumors < 200 mm^3^ for 23 days (Supplementary Figure 8B). Furthermore, treatment with the TMZ + VE822 combination resulted in a 31-day increase in survival for this model (Figure 6B) compared to TMZ alone. This combination did not result in a substantial and sustained reduction in mice body weights (Supplementary Figure 8D–8E) compared to single agents or vehicle indicating that it is well tolerated *in vivo*. These results suggest that MGMT^low^ MMR^proficient^ HR^low^ tumors are better targeted by the TMZ + ATRi combination than by single agents resulting in improved survival. Furthermore, tumors expressing low REV3L mRNA levels are better targeted with this combination even if unresponsive to TMZ as a single agent. Therefore, these *in vivo* data suggest that criteria to identify tumors sensitive to the TMZ + ATRi combination should include defects in the DDT programs (low REV3L mRNA expression), HR deficiencies and the status of MGMT and MMR.

## DISCUSSION

The clinical efficacy of TMZ is hindered by inherent and acquired resistance mechanisms. Low MGMT expression via promoter methylation is a generally accepted predictive biomarker in TMZ-treated glioblastoma and has been proposed as a useful biomarker for patient stratification [36]. Low MGMT levels in other types of cancer have resulted in the consideration of TMZ as a potential therapy beyond glioblastoma and melanoma [37]. MMR proficiency is a prerequisite for TMZ sensitivity [38]; however, MGMT expression and MMR loss of function do not account for TMZ resistance in all cases [39]. Indeed, either the loss of MGMT expression or MGMT inhibition using AZD5896 sensitized only a subset of the cell lines we analyzed to clinically relevant concentrations of TMZ (40 μM). Following TMZ exposure, we observed evidence of DNA damage in 2 MGMT^−ve^/MMR^proficient^ PDCs that differ in TMZ sensitivity; however, these responses were mostly transient and dampened in TMZ-insensitive lines (BL0479-72, as well as in MGMT^+ve^ LG0567 in the presence of AZD5896). This suggests an efficient repair of TMZ-induced DNA damage compared to persistent and acute DDR activation, cell cycle arrest and cell death, which are indicative of compromised repair in TMZ-sensitive BL0293 cells.

In BL0479-72 cells, the combination of TMZ + ATRi induced toxicity (apoptosis and the absence of recovery posttreatment withdrawal), while the combination of TMZ + ATMi resulted in less sensitization. This underscores the importance of ATR in the resistance of BL0479-72 to TMZ. ATR activation in response to TMZ has been documented and presents an attractive therapeutic target, especially in TP53-mutated cells that rely on the S and G_2_/M checkpoints for DNA damage repair [14].

The combination of TMZ + ATRi sensitized 14 TMZ-resistant lines and increased the sensitivity of 9 TMZ-sensitive lines to physiologically achievable TMZ concentrations. However, ATR inhibition did not sensitize 13 TMZ-resistant lines or increase the sensitivity of 3 TMZ-sensitive lines, even upon MGMT inhibition. Moreover, TP53 wild type status did not correlate with the modulation of TMZ responses by ATRi or lack thereof (6/24 models sensitized *de novo* or further sensitized to TMZ by ATRi have wild type TP53 and 7/13 lines not sensitized by the combination have mutant TP53). Furthermore, our data show that ATRi treatment does not overcome MGMT- and/or MMR deficiency-mediated resistance to TMZ. Indeed, these results underscore the need for determinants beyond MGMT and MMR to predict cancer cell responsiveness to the TMZ + ATRi combination.

Augmented HR activity appears to represent a novel mechanism of TMZ resistance [40]. Remarkably, the BL0293 model has a significantly low HR capacity, suggesting that profound HR deficiency might contribute to TMZ sensitivity. However, our results indicate that efficient targeting of HR either with an ATM inhibitor or interference with RAD51 or BRCA1 are not sufficient to induce TMZ responsiveness, even in the absence of MGMT. On the other hand, ATR inhibition increased the TMZ sensitivity of “HR-low” models (UWB1.289 cells or UWB1.289-BRCA1 pre-treated with B02) in the absence of MGMT. Also, RAD51 knockdown increased the efficacy of the TMZ + ATRi combination in BL0479-72 cells with a 3-fold reduction in the TMZ IC_50_ value compared to the NT shRNA cells. TMZ sensitization was achieved using a VE821 concentration that had a <5% effect on RAD51 shRNA cells, eliminating additivity as a potential cause for the enhanced effectiveness. This result suggests that TMZ-induced DNA damage, in the presence of decreased HR capacity, increases the reliance of cancer cells on ATR signaling to mediate DNA repair, thereby rendering HR deficiency a potential pre-determinant of TMZ + ATRi responsiveness. Consistently, a reduced HR capacity via RAD51 inactivation has been reported to impair replication fork progression and fork protection, leading to elevated replication checkpoint signaling [28]. Indeed, although RAD51 reduction by shRNA in BL0479-72 did not increase baseline ATR activation, it was significantly increased by TMZ treatment. By incorporating low HR capacity (via high %LOH) as a selection criterion in addition to low MGMT and MMR proficiency, we demonstrated the efficacy of the TMZ + ATRi combination in delaying tumor growth and mortality in 2 PDX models, compared to TMZ as a single agent.

CHK1 mediates several processes downstream of ATR, including the prevention of origin firing, inhibition of cell cycle progression and promoting RAD51 binding to DNA to facilitate HR [41]. CHK1 inhibition induced DSB signaling and reduced HR capacity in BL0479-72 similar to VE821. However, sensitization to TMZ was more pronounced with ATRi, suggesting the involvement of CHK1-independent ATR functions in TMZ resistance. Indeed, ATR is known to prevent replication collapse by protecting replication forks through RPA, independent of CHK1 [42]. ATR can also activate AKT in a MMR-dependent fashion as a mechanism of TMZ resistance [43]. However, the PI3K inhibitor AZD5363 failed to sensitize BL0479-72 cells to TMZ (although it induced significant toxicity as a single agent, data not shown), suggesting that AKT activation is not a mediator of resistance downstream of ATR in this model.

TLS polymerases such as Pol η, Pol κ and Pol ζ can bypass TMZ-induced O^6^-MeG lesions on DNA. Depletion of specific TLS polymerases affects cellular sensitivity to certain alkylating and crosslinking agents by increasing mutagenesis and resistance [5, 44]. Moreover, REV3L knockdown combined with ATRi was reported to enhance cisplatin cytotoxicity in sensitive and resistant non-small cell lung cancer cells [45]. Furthermore, REV3L is required for maintaining viability after replication fork stalling [46]. Therefore, low REV3L mRNA expression would create an increased reliance on ATR to maintain genomic stability and cell viability. In line with this hypothesis, we detected lower levels of REV3L mRNA expression in models sensitive to ATRi and TMZ + ATRi, compared to resistant lines. The relevance of REV3L mRNA expression in identifying TMZ + ATRi sensitive models is underscored by the significant tumor growth delay and improved survival of mice bearing low-REV3L compared to higher-REV3L TMZ-resistant PDXs, despite those having some response to TMZ as a single agent. REV3L is mutated or deleted in a subset of solid tumors [47], suggesting that TMZ + ATRi might be an attractive combination for an array of tumor types.

Recently Jackson et al. reported that TMZ further sensitizes MGMT^−ve^ cell lines to ATRi and pinpointed MGMT negativity as a biomarker for predicting the effectiveness of TMZ + ATRi in lines that are moderately sensitive to TMZ [21]. We report that the combination of ATRi with TMZ greatly increases the efficacy of this agent in MGMT^low/−ve^ MMR^proficient^ models of various cancer origins and sensitizes a panel of resistant lines by preventing the repair of TMZ-induced DNA damage. However, we have further identified HR defects and DDT deficiencies (low REV3L levels) as additional predictors/biomarkers for the efficacy of this combination. Indeed, a PDX model selected based on these criteria (MGMT^low^/MMR^proficient^/LOH^high^/REV3L^low^) showed increased efficacy for the TMZ + ATRi combination. This suggests that ATR is a good target in the context of TMZ treatment for tumor types beyond glioblastoma and advanced melanoma with specific molecular determinants that can be used to identify patients that might benefit from this combination.

## MATERIALS AND METHODS

### Cell lines and reagents

Patient-derived cell lines (PDC) and NCI-60 lines were obtained from the PDMR (https://pdmr.cancer.gov) and the NCI Developmental Therapeutics Program (DTP). Cells were grown in RPMI-1640 (Quality Biologicals) with 10% fetal bovine serum (FBS) and L-glutamine. AmpFLSTR^®^ Identifiler^®^ (Applied Biosystems) testing with PCR amplification and mycoplasma testing is conducted to confirm consistency with the published Identifiler^®^ STR profile [48] and mycoplasma-free status of each given line upon deposition to the repository. The cells were kept in continuous culture at NCI for < 20 passages. UWB1.289 ± BRCA1 (ATCC) were grown in 50% RPMI-1640, 50% Mammary Epithelial Growth Medium (Lonza), 3% FBS and the BRCA1 line media also contained 200 μg/mL G418 (Gibco). HCT116, HCT116 + Chr3, DLD-1 and DLD-1 + Chr2 (a gift from Dr. Thomas Kunkel; NIEHS) were grown in DMEM/F12 (Gibco) with 10% FBS and the media for the chromosome-reconstituted lines also contained 400 μg/mL G418. All cell lines were maintained at 37°C, 5% CO_2_ and 95% humidity. Mycoplasma testing was performed using MycoAlert™ PLUS Mycoplasma Detection Kit (Lonza).

TMZ, ATRi (VE821, VE822/VX970/M6620, AZD6738), CHK1 inhibitors (LY2606368, MK8776, LY2606318), ATM inhibitor (KU55933), MGMT inhibitor (AZD5896) and roscovitine were obtained from the Drug Synthesis and Chemistry Branch, DTP, NCI. B02 (RAD51 inhibitor) and NU7441 (DNA-PK inhibitor) were purchased from EMD Millipore and Tocris, respectively. All compounds were resuspended in DMSO and stored at –80°C, except TMZ, which was prepared fresh in DMSO prior to treatment. When necessary, AZD5896 was added 2 h prior to treatment with other agents.

### Cell proliferation and prolonged exposure/washout assay

Cells were seeded at ~2,000 cells/well in 100 μl in ViewPlate-96 plates (PerkinElmer), treated 24 h later with TMZ ± inhibitors for a total volume of 200 μl (final DMSO concentration < 0.2%) and incubated for 96 h or as specified. Then, plates were cooled to room temperature (RT) and CellTiter-Glo^®^ (CTG, Promega) was added at 100 μl/well, the plates were shaken on an orbital shaker for 2 min and incubated in the dark for 10 min. Luminescence was measured with a 1 s integration time using an infinite M1000 microplate reader (Tecan). The CTG results were confirmed by the sulforhodamine B (SRB) assay, which was performed as described previously [49]. A minimum of three independent experiments were performed. Results of cell lines and PDCs were plotted as a heat map of % inhibition or [100- % T/V at each dose calculated] as follows: 100- % inhibition at dose x = 100 × (average blanked luminescence from wells treated with dose x)/(average blanked luminescence from wells treated with vehicle). Results were plotted using the heat map function in excel with a color gradient where blue backgrounds have the lowest treatment induced inhibition while red backgrounds indicate the most acute treatment induced inhibition. Results of BL0479 treated with TMZ and various inhibitors are presented as line graphs of % T/V: 100 × (average blanked luminescence from wells treated with dose x)/(average blanked luminescence from wells treated with vehicle), IC_50_ values were calculated using the equation Y = a × X + b, IC_50_ = (0.5 − b)/a with log transformed doses. For prolonged exposures, PDCs were seeded at ~1,000 cells/well in 200 μl in 6 replicates in 48-well plates (Corning 3548) and treated 24 h later with DMSO, TMZ, VE821, AZD6738 or VE822 or combination of TMZ + ATRi for a total volume of 400 μl/well for 96 h. After 96 h, the medium was replaced with agent-free medium for 3 replicates/treatment. The plates were processed for SRB at the indicated times.

### Homologous recombination DRGFP assay

To measure HR capacity, PDCs were co-transfected with pDR-GFP + pCBASceI (a gift from Dr. Maria Jasin, Addgene #26475 and #26477) or pDR-GFP + pFUGW-RFP (transfection efficiency control) (1:1) for a total of 2.5 μg DNA using lipofectamine LTX with Plus Reagent (Invitrogen) according to the manufacturer’s protocol in OptiMEM (Gibco) in 6-well plates (Corning 3506). Six hours post-transfection, media was replaced with media containing vehicle or inhibitors. Cells were incubated for an additional 48 h, collected, and analyzed for GFP and RFP expression using a FACSCalibur cytometer with CellQuest Pro software (BD Biosciences).

To evaluate the effects of DDR inhibitors on HR capacity, PDCs were transfected with pDR-GFP and selected with 1–10 μg/ml puromycin 48 h later. Post-selection, PDR-GFP stable lines were seeded in 6-well plates, allowed to attach overnight, and transfected with pCBASceI or pFUGW-RFP using lipofectamine LTX as above. Six hours post-transfection, media was replaced with media containing vehicle or inhibitors. Cells were incubated for an additional 72 h, collected and analyzed for GFP expression using a FACSCalibur cytometer with CellQuest Pro software (BD Biosciences).

### Cell cycle analysis

Cells were seeded in 60 mm dishes (Corning 430166) (75,000 cells/well) and treated with vehicle or drugs 24 h later. Following the indicated drug exposure times, the cells were pulsed with 10 μM bromodeoxyuridine (BrdU; Sigma) for 90 min, trypsinized, washed with 1x PBS, fixed in ice-cold 70% ethanol, denatured with 2 N HCl for 25 min at RT, followed by inactivation with 0.1 M sodium borate, pH 8.5. Cells were then incubated with anti-BrdU FITC (Biolegend, Clone 3D4, 364104) (5 μl in 100 μl cells suspended in 0.2% BSA in PBS) for 20 min at RT, spun down then resuspended in 500 μl PI/RNase staining buffer (BD Biosciences, 550825) for 15 min both in the dark. Cell cycle analysis was performed on a FACSCalibur cytometer/CellQuest Pro.

### Apoptosis assay

Cells were seeded in 60 mm dishes (Corning CLS430196) and treated 24 h later with TMZ, VE821, AZD6738 or combinations for 72 h, then processed using FITC Annexin V Apoptosis Detection Kit I with propidium iodide (PI) (BD biosciences) following the manufacturer’s protocol. Data were acquired using a FACSCalibur cytometer/CellQuest Pro.

### MGMT overexpression vector cloning

The human MGMT cDNA sequence was amplified using Phusion^®^ High-Fidelity DNA Polymerase (NEB) from the Myc-DDK-tagged MGMT cDNA template (Origene, NM_002412) and cloned into the lentiviral vector pCDH-pGK-puro using BamHI/EcoRI sites.

### MGMT overexpression and RAD51 knockdown

Target cells were spinoculated at a density of 2 × 10^5^/well in 6-well plates in suspension with 1 mL of lentiviral suspension of MGMT or RAD51 shRNA plasmids (Supplementary Materials) with 8 μg/mL polybrene followed by puromycin selection (1 μg/ml) 72 h later, for 1 week.

### RT-qPCR

RNA was extracted using the RNAeasy Kit (Qiagen) and reverse-transcribed with the High-Capacity cDNA Reverse Transcription Kit (Applied Biosystems). Quantitative PCR was performed in technical triplicates using Fast SYBR™ Green Master Mix or TaqMan™ Fast Universal PCR Master Mix (2X), no AmpErase™ UNG (Thermofisher) with primers for MGMT, RAD51, Actin, probes for REV1, REV3L, REV7, POLH, POLK, RRM2, and GAPDH (Supplementary Table 3).

### Western blot

Protein lysates were prepared in lysis buffer (50 mM TRIS-HCl pH 7.5, 150 mM NaCl, 10% glycerol, 25 mM NaF, 5 mM β-glycerophosphate, 1 mM sodium orthovanadate, 2 mM EGTA, 2 mM EDTA, 1% NP40) with a cOmplete™, Mini Protease Inhibitor Cocktail Tablet (Roche). Forty μg protein lysates were resolved on 4–20% Criterion™ TGX™ gels (Bio-Rad) and transferred onto nitrocellulose membranes using a Trans-Blot Turbo (Bio-Rad). Antibodies (listed in Supplementary Materials) were diluted in 5% BSA (phospho-proteins) or 5% nonfat milk (total proteins) in Tris-Buffered Saline, 0.1% Tween^®^ 20 (TBST) and incubated at 4°C overnight. After incubation with HRP-conjugated secondary antibodies and TBST washes, proteins were detected using SuperSignal™ West Pico PLUS chemiluminescent substrate (Thermofisher) and chemiluminescence was captured using a Chemidoc MP (Bio-Rad).

### Immunofluorescence

BL0479-72 and BL0293 cells were seeded in Nunc™ Lab-Tek™ II CC2™ 4-well chamber slides (10,000 cells/well) (Thermo Scientific) and treated 24 h later as indicated. Slides were fixed overnight in ice-cold 70% ethanol at –20°C, air dried, permeabilized using 1x PBS + 0.1% Tween20, blocked with 2% normal goat serum in Odyssey^®^ Blocking Buffer (PBS) (LI-COR) then incubated with Alexa 488-tagged γH2AX (EMD 05-636-AF488, 1:100) for 1 hr at RT, washed and mounted with ProLong Gold antifade (Invitrogen). Images were captured by confocal microscopy using a Nikon 90i at 20x. Quantification was performed using Definiens Tissue Studio software with nuclear area positive analysis as described [50].

### *In vivo* drug study

Human bladder carcinoma patient-derived xenograft (PDX) model BL0382-F1232 and squamous cell lung carcinoma PDX LG0520-F434 were purchased from Jackson Laboratories. PDX tumor fragments (~1 mm^3^) were implanted subcutaneously in sex-matched NSG mice (NOD.Cg-PrkdcscidIl2rgtm1Wjl/SzJCr); tumors were staged to 150 mm^3^ then mice were randomized to treatment groups: 4–5 mice/treatment group and 15 mice for vehicle were used for model BL0382-F1232, while 6 mice/treatment group and 16 mice for vehicle were used for LG0520-F434 using Study Director (Studylog Systems, Inc.).

Treatment groups received 2 cycles of orally-administered: vehicle (10% Vitamin E/TPGS in water: QDx4, 3 days rest, QDx4; Klucel-hydroxypropylcellulose: QDx5, 2 days rest); TMZ 50 mg/kg (in Klucel, hydroxypropylcellulose, QDx5, 2 days rest; VE822 45 mg/kg (in 10% Vitamin E/TPGS in water: QDx4, 3 days rest) or the combination of TMZ and VE822 in the schedules described. Tumor volumes and body weights were measured twice a week. Data were analyzed using multiple *t*-tests, and statistical significance was determined using the Holm-Sidak method, with α = 0.05. Each entry/mouse/day was analyzed individually, without assuming a consistent SD.

PDX model identities were confirmed by short tandem repeat profiling (AmpFlSTR Identifiler PCR Amplification Kit) and reviewed at every passage to confirm histology and human DNA: murine DNA content to confirm integrity [51].

Animal care was provided in accordance with procedures outlined in the “Guide for Care and Use of Laboratory Animals” (National Research Council; 1996; National Academy Press, Washington, D.C.), and studies were conducted under an approved Animal Care and Use Committee protocol.

### Statistical analyses

Descriptive statistics including mean, SD, SEM, and Student *t*-test were calculated with GraphPad Prism 8. A two-tailed Student’s *t*-test was used for gene expression comparisons with *p* < 0.05 considered significant. Overall synergy values were calculated as described in [52]. Briefly, data from the CTG assay with combination treatments performed in a 5 × 6 matrix plus TMZ and inhibitors alone were used to calculate a theoretical and experimental response surface based on Bliss independence log synergy similar to the MacSynergy II software analysis. The theoretical surface was constructed using the dissimilar site assumption [Z = X + Y(1 − X)] at each of the drug combinations utilizing the data from the dose responses of the individual drugs. The experimental surface was constructed using the data obtained from the drug combinations. After both surfaces were constructed, the theoretical surface was subtracted from the experimental surface to reveal regions of synergy and antagonism. Positive values were summed together to give the overall synergy value (μM^2^%) across the response surface. Degrees of synergy were based on Smee *et al.* [53] with >100 μm^2^ unit % calculated values in a positive direction defined as strong synergy. Western blot densitometry was done using ImageJ (NIH) gel analysis function from at least 3 different blots.

## SUPPLEMENTARY MATERIALS



## Abbreviations

TMZ: Temozolomide
MGMT: O^6^-Methylguanine-DNA methyltransferase
MMR: mismatch repair
ATR: Ataxia telangiectasia and Rad3 related
ATRi: ATR inhibitor(s)
ATM: Ataxia telangiectasia mutated
DSB: double-strand break
DDR: DNA damage response
CHK1/2: Checkpoint kinase 1/2
TLS: Translesion
^−ve^: negative
^+ve^: positive
HR: homologous recombination
DDT: DNA damage tolerance
PDMR: NCI Patient-Derived Models Repository
SRB: Sulforhodamine B
CTG: CellTiter-Glo
PI: propidium Iodide
