# Supplementary material for: ATR inhibition reverses the resistance of homologous recombination deficient MGMTlow/MMRproficient cancer cells to temozolomide
Source: Oncotarget. 2021 Oct 12;12(21):2114–30. doi: 10.18632/oncotarget.28090 (PMC8522839; doi:10.18632/oncotarget.28090)
Supplement: Supplementary file 1 [file oncotarget-12-2114-s001.pdf]

ATR inhibition reverses the resistance of homologous recombination deficient MGMT^{low}/MMR^{proficient} cancer cells to temozolomide

SUPPLEMENTARY MATERIALS

Antibodies

The antibodies used are: pATR^{T1989} (Pharmacodynamic Assay Development and Implementation Section; PADIS, DCTD, NCI [48], pATM^{S1981} (Cell Signaling, #13050, 1:500), pTIF1β^{S824} (Cell Signaling, #4127, 1:1000), PARP (Cell Signaling, #9542, 1:1000), pCHK1^{S345} (Cell Signaling, #2341, 1:1000), pChk2^{T68} (Cell Signaling, #2661, 1:1000), pCDK1^{Y15} (Cell Signaling, #9111, 1:1000), pHistone H3^{S10} (Cell Signaling, #9701, 1:1000), pRPA$^{S4/8}$ (Bethyl Laboratories, A300-245A, 1:1000), ATR (Cell Signaling, #2790, 1:1000), ATM (Cell Signaling, #2873, 1:1000), TIF1β (Cell Signaling, #4123, 1:1000), CHK1 (Cell Signaling, #2360, 1:1000), CHK2 (Cell Signaling, #2662, 1:1000), MGMT (Cell Signaling, #2739, 1:1000), γH2AX (clone JBW301, Millipore 05-636, 1:1000), β-actin (EMD Millipore, clone C4 MAB1501, 1:10000), GAPDH (1:1000) and HRP-conjugated anti-mouse (Santa Cruz) and anti-Rabbit (Cell Signaling).

Lentiviral preparation

Eighty percent confluent HEK-293T (Clontech) in 10-cm plates (Corning CLS430167) were transfected with PCDH-PGK-Puro-Myc-DDK-tagged MGMT or RAD51 shRNA plasmids (RHS4533-EG5888, GE-Dharmacon) using Polyethyleneimine (PEI) at a 3:1 ratio with 8 µg total DNA mixture (4:2:1) of gene of interest, psPAX2 (Didier Trono; Addgene #12260) and pCMV-VSV-G (Robert Weinberg; Addgene #8454) in serum-free DMEM (Gibco). The media was replaced with DMEM 10% FBS 6 h later. The supernatant containing lentiviral particles was collected 48 h later, filtered through a 0.45 µm PES filter, aliquoted and stored at −80°C.

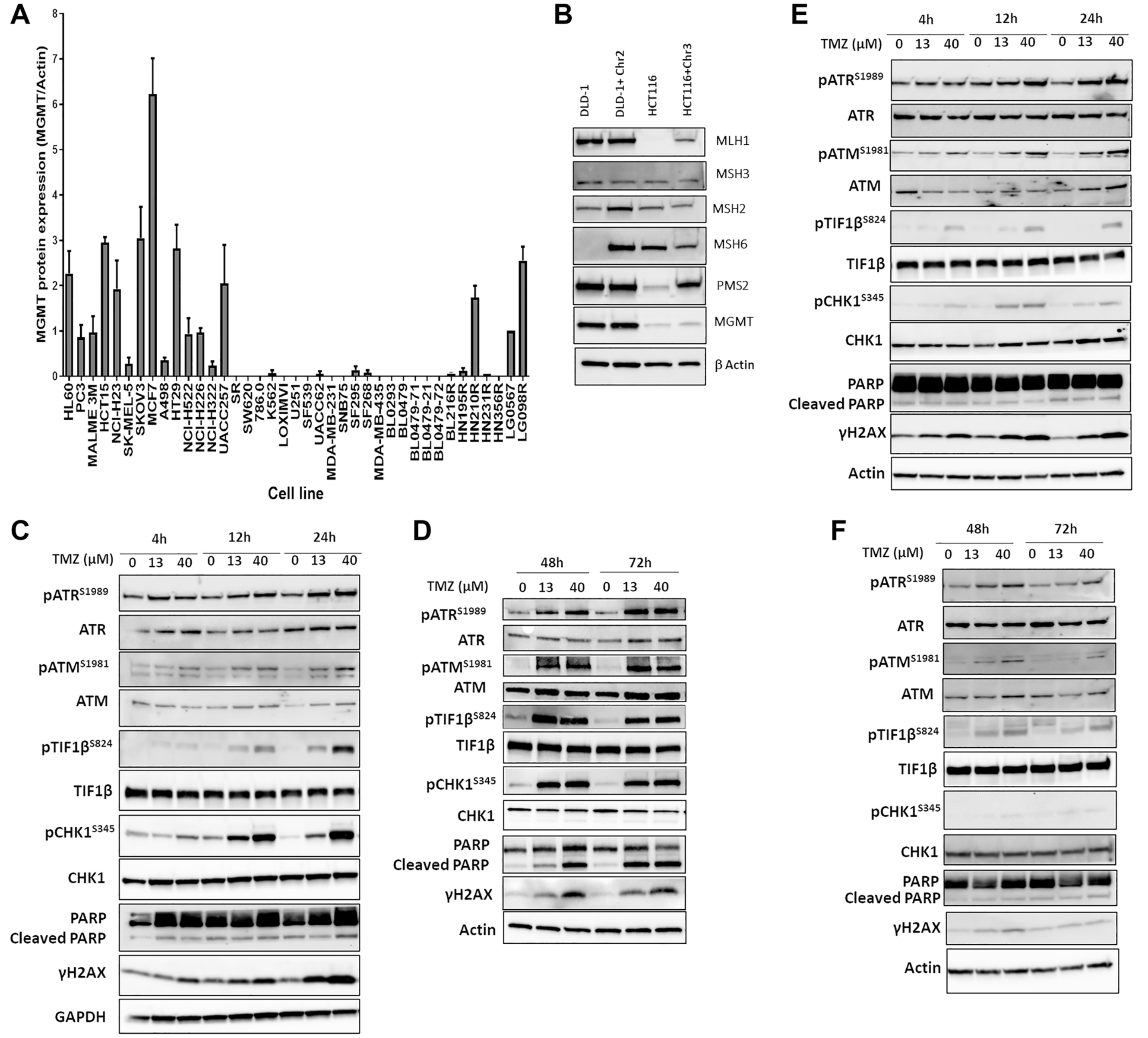

Supplementary Figure 1: (**A**) MGMT protein expression was measured by Western blot and normalized to actin levels. An average of two independent experiments are plotted ± SEM. (**B**) A Western blot shows the expression of MMR proteins and MGMT in the MMR-deficient cell lines DLD-1 and HCT116, along with their reconstituted counterparts DLD-1 + Chromosome (Chr2) and HCT116 + Chr3. (**C**) DDR signaling was assessed in BL0293 cells treated with TMZ (13 μM or 40 μM) for 4 h, 12 h, 24 h, as well as (**D**) 48 h and 72 h. (**E**) DDR signaling was assessed in BL0479-72 cells treated with TMZ (13–40 μM) for 4 h, 12 h, 24 h, as well as (**F**) 48 h and 72 h.

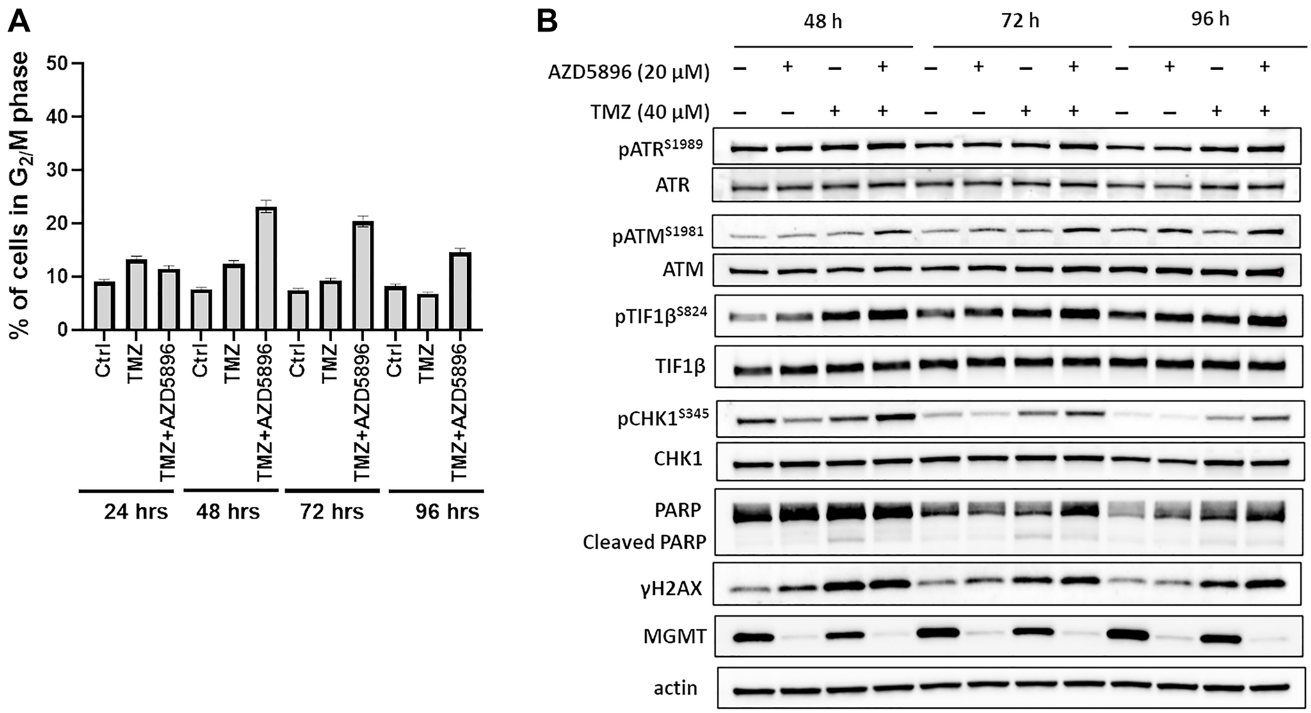

Supplementary Figure 2: (**A**) LG0567 lung adenocarcinoma cells were pre-treated ± AZD5896 (20 µM) for 2 h followed by vehicle or TMZ (40 µM) treatment for the indicated times, pulsed with BrdU and processed for cell cycle analysis, results are the mean of 2 independent experiments ± SEM. (**B**) DDR signaling in LG0567 ± AZD5896 ± TMZ at the indicated time points was assessed by Western blot.

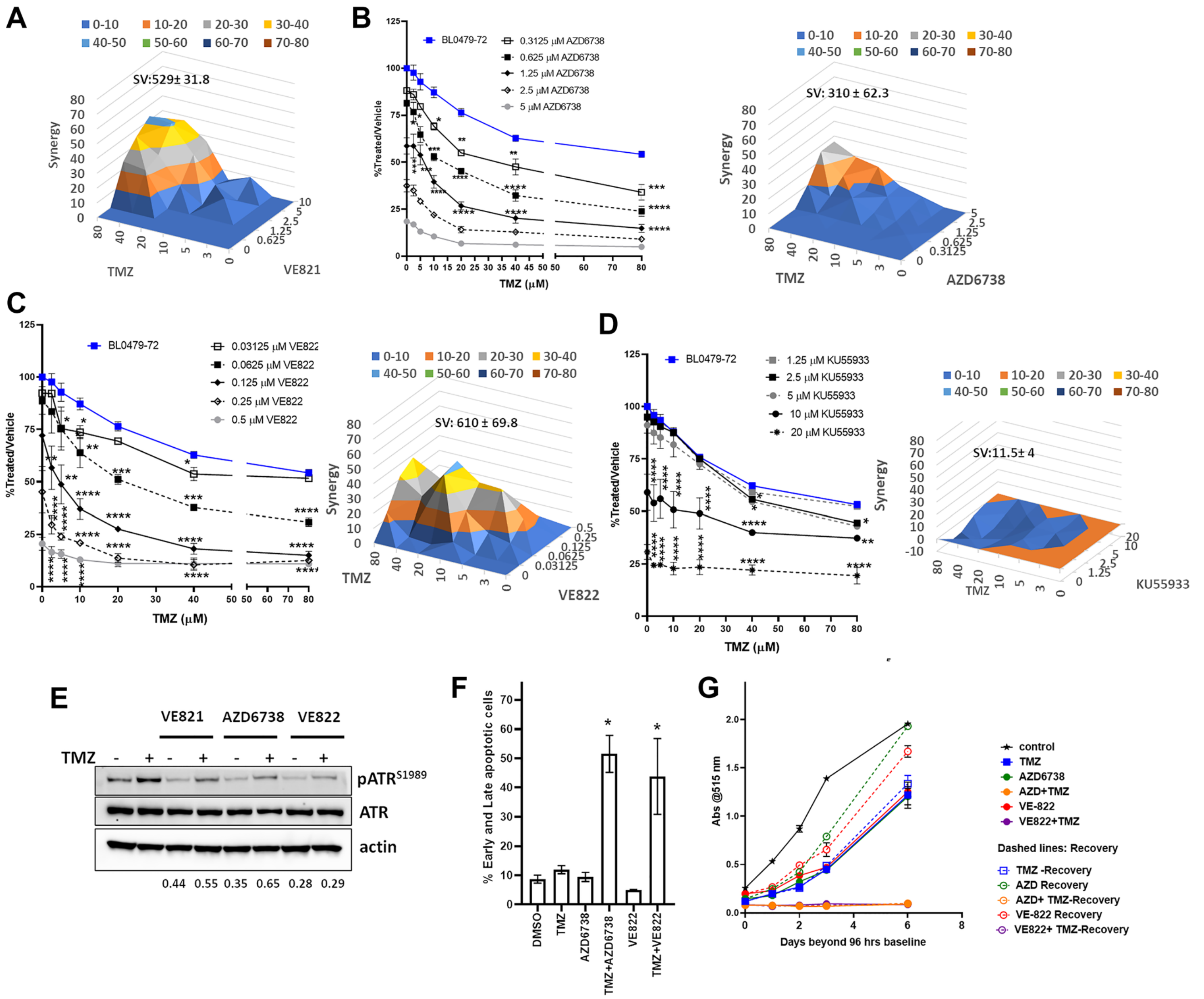

Supplementary Figure 3: (**A**) 3D model of BL0479-72 cells treated for 96 h with 6 concentrations of TMZ (0–80 µM) in combination with 5 concentrations of the ATRi VE821. Line graphs (%T/V) and 3D model (right panels) of BL0479-72 cells treated for 96 h with 6 concentrations of TMZ (0–80 µM) in combination with 5 concentrations of the ATRi (**B**) AZD6738 (0–5 µM), (**C**) VE822 (0–0.5 µM), or (**D**) the ATM inhibitor KU55933 (0–20 µM). Cell proliferation was quantified by CellTiter-Glo and results shown are averages of 3 independent experiments. $^{*}p < 0.05$, $^{**}p < 0.01$, $^{***}p < 0.001$, $^{****}p < 0.0001$ compared to same dose of TMZ as a single agent. The expected synergy value (SV) for each drug combination is 0, assuming no synergy or antagonism, and values >100 suggest synergy. The SV is shown as the mean ± SEM from 3 independent experiments. (**E**) BL0479-72 cells were treated for 24 h with TMZ (40 µM) ± VE821 (1.25 µM), AZD6738 (0.5 µM) or VE822 (0.125 µM) and lysates were probed for pATR^{S1989}, total ATR and actin. Densitometry of ATR activation (pATR/ATR) normalized to vehicle-treated cells +/− TMZ is shown below the blots (**F**) BL0479-72 were treated for 72 h with TMZ (40 µM) ± AZD6738 (1 µM) or VE822 (0.1 µM) and processed for annexin V/PI staining. The percentage of early and late apoptotic cells are plotted as the mean ± SEM of 3 independent experiments ($^{*}p < 0.05$ compared to DMSO or TMZ, AZD6738 and VE822 as singe agents). (**G**) BL0479-72 were treated with TMZ (40 µM) ± VE822 (0.1 µM) or AZD6738 (0.5 µM). After 96 h, the treatments were replaced with inhibitor-free medium (Recovery) or retained, and proliferation was assessed by SRB at indicated times. The data are plotted as mean ± SEM from 2 independent experiments.

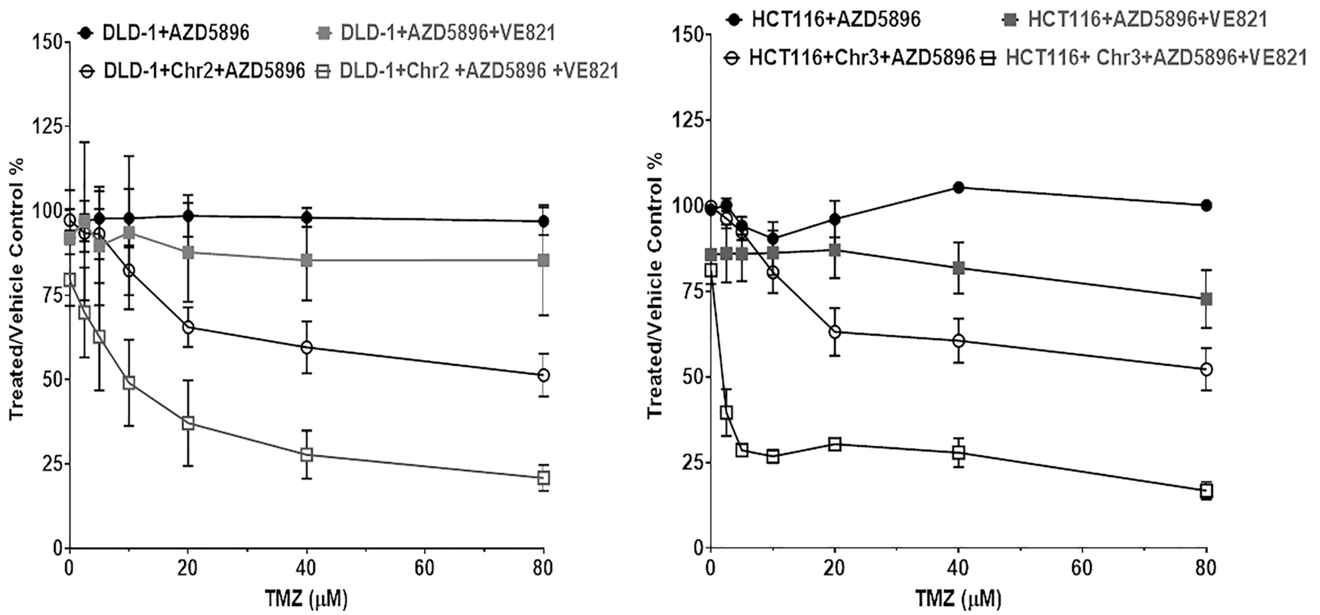

Supplementary Figure 4: DLD-1 (*Left*) and HCT116 (*right*) cells and their Chr2 and Chr3 reconstituted counterparts were pre-treated with AZD5896 (10 μM) for 2 h, followed by treatment with TMZ (0–80 μM) ± 2.5 μM VE821 for 96 h. Percent proliferation relative to the vehicle control was quantified by CTG and is plotted as the mean ± SEM of 2 independent experiments ($^{*}p < 0.05$, $^{**}p < 0.01$, $^{***}p < 0.001$, $^{****}p < 0.0001$ compared to no VE821).

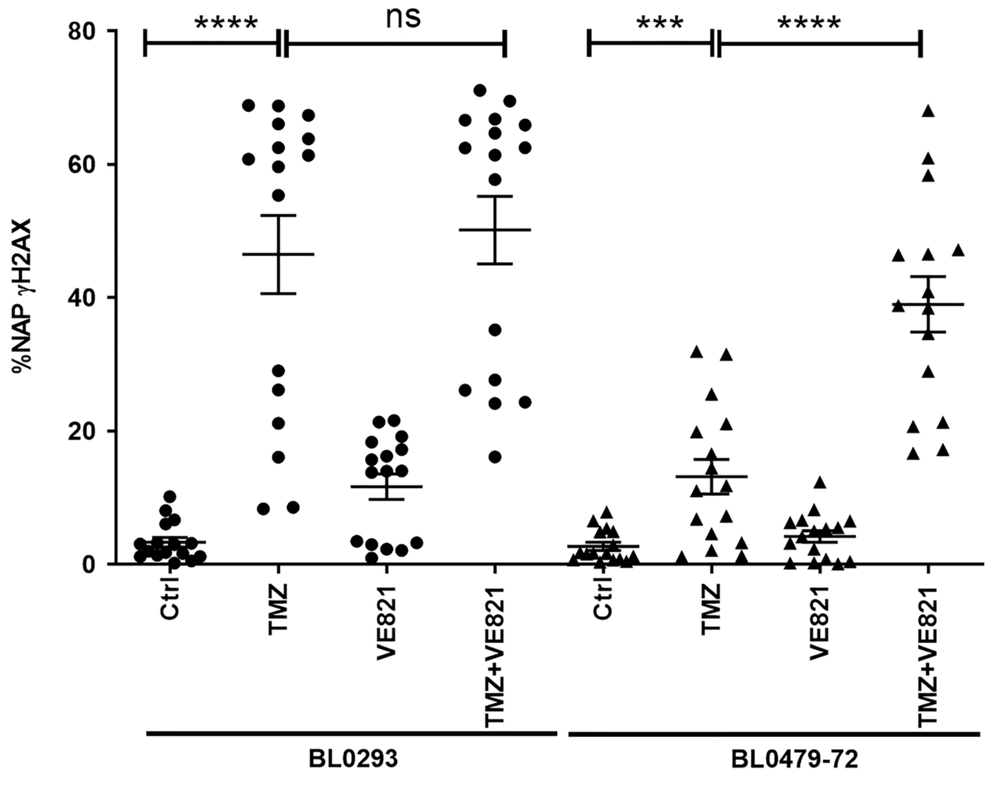

Supplementary Figure 5: BL0293 and BL0479-72 cell lines were treated with vehicle or TMZ (40 μM) ± VE821 (1 μM) for 48 h. The percent nuclear area positive (NAP) was measured for γH2AX on stained slides from 3 independent experiments with > 1000 cells scored in total and are shown with bar representing mean ± SEM (ns, not significant, $^{***}p < 0.001$, $^{****}p < 0.0001$).

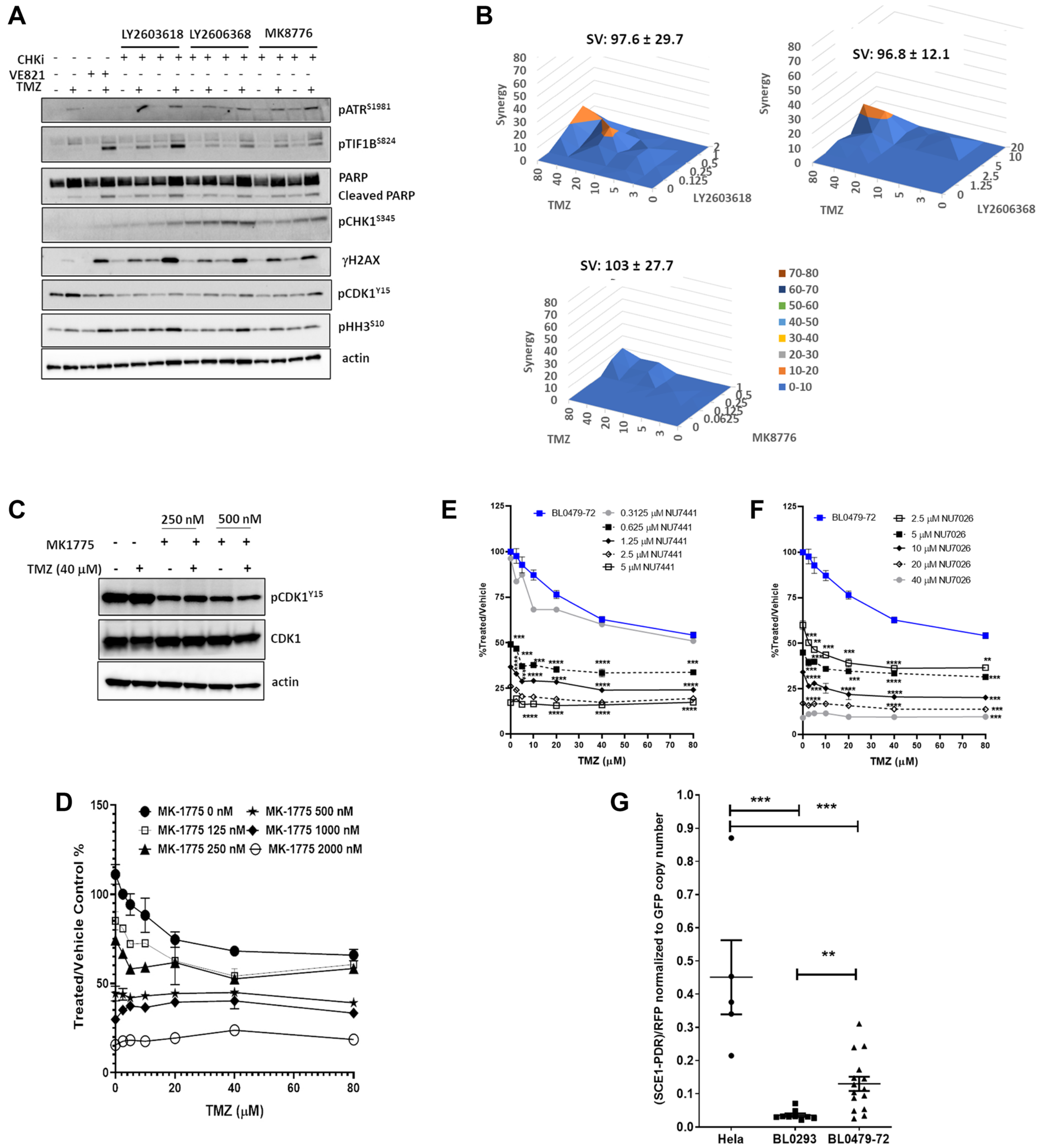

Supplementary Figure 6: (**A**) BL0479-72 cells were treated for 72 h with vehicle, TMZ (40 μM), VE821 (1 μM) or the CHK1i LY2603618 (0.25, 0.5 μM), LY2606368 (2.5 and 5 nM) and M8776 (0.25 and 0.5 μM) as single agents or combinations. DNA damage signaling was evaluated by Western blot (**B**) 3D response plots show the results of BL0479-72 cells treated with combinations of TMZ and LY2603618, LY2606368 or M8776 (at indicated concentrations. The expected synergy value (z-axis) for each drug combination is 0, assuming no synergy or antagonism, and values above the plane represent additivity/synergy with >100 suggesting synergy. The volume of synergy (SV) for each combination is calculated as the mean ± SEM of at least 3 independent experiments. (**C**) BL0479-72 cells were treated with TMZ (40 μM) and MK1775 (250 or 500 nM) for 24 h before pCDK1^{Y15}, total CDK1 and actin were assessed by Western blot. (**D**) Proliferation was assessed 96 h after BL0479-72 cells were treated with a combination of TMZ (0–80 μM) and MK-1775 (0–2 μM). (**E**–**F**) Line graphs (%T/V) of BL0479-72 cells treated for 96 h with 6 concentrations of TMZ (0–80 μM) in combination with 5 concentrations of the DNAPKi (E) NU7441 (0–5 μM), (F) NU7026 (0–40 μM). Cell proliferation was quantified by CellTiter-Glo and results shown are averages of 3 independent experiments. $^{*}p < 0.05$, $^{**}p < 0.01$, $^{***}p < 0.001$, $^{****}p < 0.0001$ compared to same dose of TMZ as a single agent. (**G**) The HR capacities of BL0293, BL0479-72 and HeLa were assessed by the DRGFP/SceI assay. Individual values are shown, and error bars indicate the mean ± SEM of at least four independent experiments ($^{**}p < 0.01$ and $^{***}p < 0.001$).

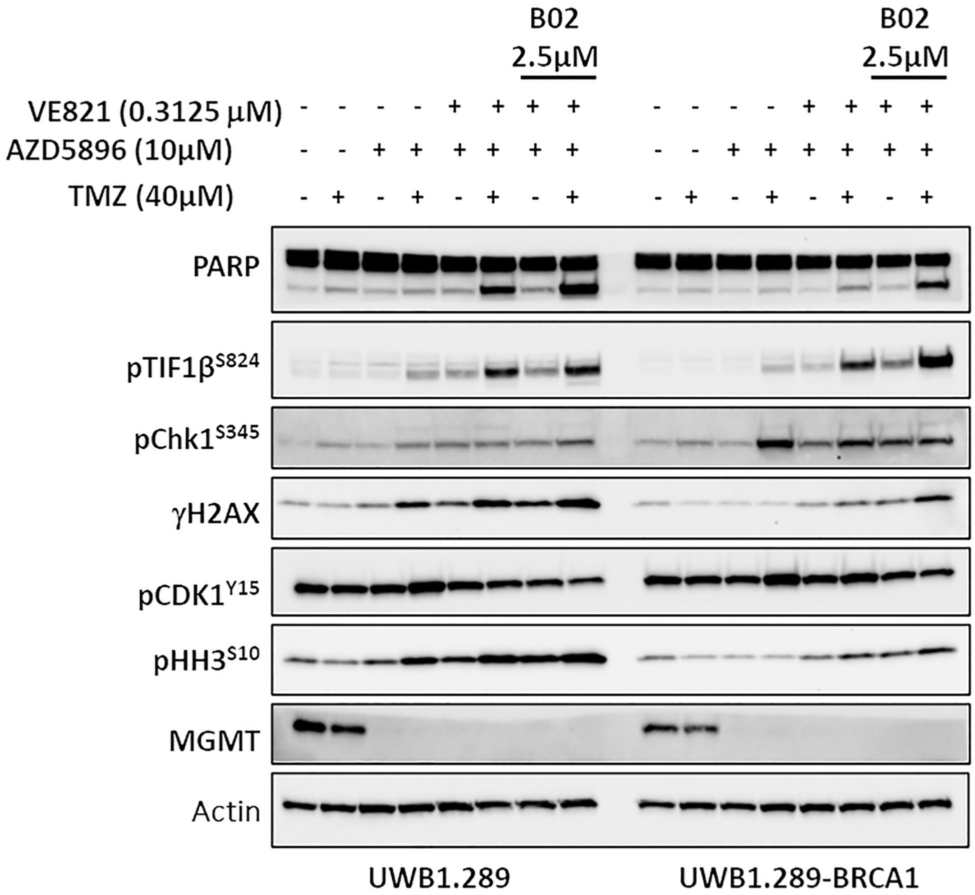

Supplementary Figure 7: UWB1.289 (*left*) and UWB1.289-BRCA1 (*right*) cells were pretreated ± AZD5896 (10 μM) for 2 h, followed by TMZ (40 μM) ± VE821 (0.3125 μM). After 72 h DDR signaling was assessed by Western blot. For the lanes marked with B02, the cells were pretreated with B02 (2.5 μM) for 1 h prior to TMZ addition.

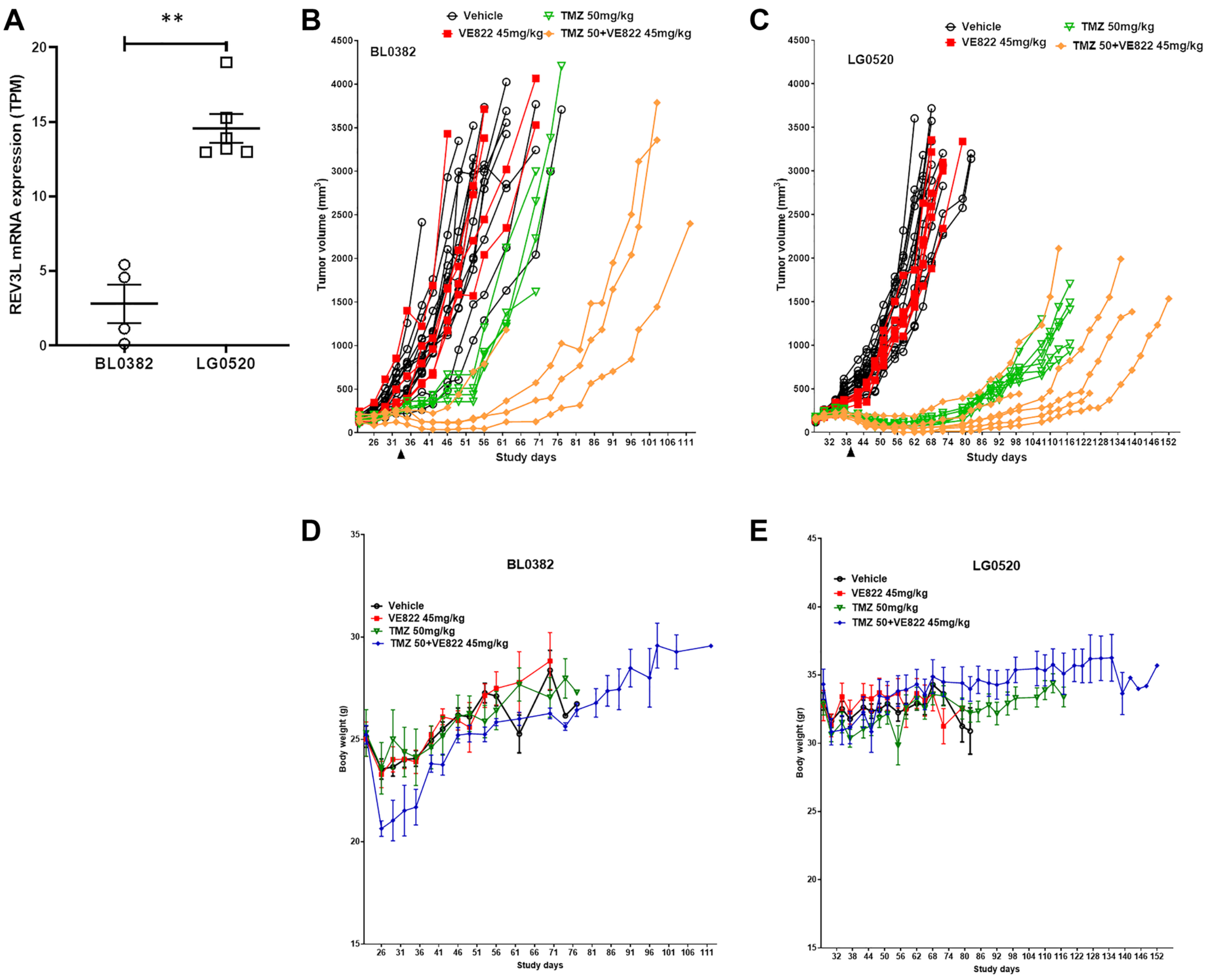

Supplementary Figure 8: (**A**) REV3L mRNA expression measured by RNASeq in BL0382 and LG0520-derived PDXs (passage 2–4). Individual values (each data point represents value from a distinct tumor fragment from a distinct mouse) are shown and error bars indicate mean ± SEM ($^{**}p < 0.01$). Mice harboring (**B**) BL0382 or (**C**) LG0520 tumor fragments were treated with vehicle, TMZ (50 mg/kg; QDx5, 2-day rest, QDx5), VE822 (45 mg/kg; QDx4, 3-day rest, QDx4) or TMZ + VE822. Treatments were administered between (B) days 22 and 33 or (C) days 28 and 39. Tumor volume measurements of individual mice are shown, and the arrowheads depict the end of treatment. (**D–E**) Average body weights in grams of mice bearing BL0382 and LG0520 tumors.

Supplementary Table 1: The patient-derived cell lines used in this study

PDC name	Cancer type	Histopathology	P53 status	MGMT status
BL0293-F563-PDC	Urothelial/bladder	Sarcomatoid transitional cell carcinoma	p.R248Q, 100%	Negative
BL0479-F1894-PDC (21/71/72)	Urothelial/bladder	Transitional cell carcinoma	p.R280T, 100%	Negative
889142-216-R-J1-PDC	Urothelial/bladder	Squamous cell carcinoma	p.G266Dfs*79, 100%	Negative
328373-195-R-J1-PDC	Head and neck	Squamous cell carcinoma	p.R342*l, 96%	Positive
959717-210-R-PDC	Head and neck	Lip/oral cavity squamous cell carcinoma	p.R280T, 99%	Positive
981375-231-R-J1-PDC	Head and neck	Lip/oral cavity squamous cell carcinoma	WT	Negative
929823-356-R-J2-PDC	Head and neck	Lip/oral cavity squamous cell carcinoma	p.R248W, 65%	Negative
LG0567-F671-PDC	Lung	Non small cell Lung adenocarcinoma	p.R273C, 100%	Positive
349418-098-R-PDC	Lung	Non small cell Lung adenocarcinoma	WT	Positive

The listed PDC names are identical to the distribution lot names provided by the PDMR.

Supplementary Table 2: The established cell lines used in this study with their p53, MGMT and MMR status

Cell line	Disease	p53 status	MGMT status	MSI
U251	Astrocytoma	p.R273H, 100%	Negative	No
SF295	Glioblastoma	p.R248Q, 100%	Negative	No
SNB75	Glioblastoma	p.E258K, 100%	Negative	No
SF539	Gliosarcoma	p.R342fs*3, 100%	Negative	No
SF268	Astrocytoma	p.R273H, 100%	Negative	No
NCIH23	Lung adenocarcinoma	p.M246I, 100%	Positive	No
NCIH522	Lung adenocarcinoma	p.P191fs*56, 100%	Positive	No
NCIH322M	lung adenocarcinoma	p.R248L, 100%	Positive	
NCIH226	Pleural epithelioid mesothelioma	Disputed	Positive	No
MDA-MB 435	Amelanotic melanoma	p.G266Q, 50%	Negative	
LOXIMVI	Amelanotic melanoma	WT	Negative	No
MALME 3M	Melanoma	WT	Positive	No
UACC62	Melanoma	WT	Negative	No
UACC257	Melanoma	WT	Positive	No
SKMEL5	Cutaneous melanoma	WT	Positive	No
A498	Renal cell carcinoma	WT	Positive	No
786.0	Renal cell carcinoma	p.P278A, c.560-2A>G 50%	Negative	No
HT29	Colon adenocarcinoma	p.R273H, 100%	Positive	No
SW620	Colon adenocarcinoma	p.R273H, 100%	Negative	No
HCT15	Colon adenocarcinoma	p.S241F, 50%	Positive	YES
HL60	Acute myeloid leukemia	Deleted	Positive	No
SR	Anaplastic large cell lymphoma	WT	Negative	No
K562	Chronic myeloid leukemia	p.Q136fs*13, 100%	Negative	No
MCF7	Invasive ductal carcinoma	WT	Positive	No
MDA-MB231	Breast adenocarcinoma	p.R280K, 50%	Negative	No
SKOV3	Ovarian serous cystadenocarcinoma	p.S90fs*33, 50%	Positive	Yes
PC3	Prostate carcinoma	p.K139fs*31, 100%	Positive	No

MSI: Microsatellite instability or MMR deficiency.

Supplementary Table 3: Primers and probes used for SYBR green and Taqman PCR

Gene	Primer/Probe	Source
MGMT	F:CTGGCCGAAACTGAGTATGT<br>R:GGACACTGCCACTTCCTTTA	
RAD51	F:TGTTGTGACTGCCAGGATAAA<br>R: GGTAGATGGTGAAGGGCTAATG	IDT
Actin	F:CCTGGCACCCAGCACAAT<br>R:GCGGATCCA CACGGAGTACT	
REV1	Hs01019768_m1	
REV3L	Hs00161301_m1	
REV7	Hs01057448_m1	
POLH	Hs00197814_m1	Applied Biosystems
POLK	Hs00211965_m1	
RRM2	Hs00357247_g1	
GAPDH	Hs99999905_m1	
